# FTO Suppresses Dental Pulp Stem Cell Senescence by Destabilizing *NOLC1* mRNA

**DOI:** 10.3390/biom15111627

**Published:** 2025-11-19

**Authors:** Bingrong Li, Mi Xu, Junjun Huang, Rong Jia

**Affiliations:** State Key Laboratory of Oral & Maxillofacial Reconstruction and Regeneration, Key Laboratory of Oral Biomedicine Ministry of Education, Hubei Key Laboratory of Stomatology, School & Hospital of Stomatology, Wuhan University, Wuhan 430072, China

**Keywords:** dental pulp stem cells, NOLC1, FTO, cellular senescence

## Abstract

Cellular senescence is an intricate process that severely restricts stem cell function. The N6-methyladenosine (m^6^A) eraser, fat mass and obesity-associated (FTO) protein control several aspects of stem cell fate, including differentiation, self-renewal, and senescence. However, the role of FTO in dental pulp stem cell (DPSC) senescence has not yet been elucidated. This study aimed to explore the role of FTO in DPSC senescence. FTO expression decreases during DPSC senescence. FTO depletion inhibited DPSC proliferation, accelerated senescence, and increased reactive oxygen species (ROS) levels. FTO overexpression reduced DPSC senescence, enhanced proliferation, and decreased ROS accumulation. RNA sequencing demonstrated that FTO knockdown inhibited ribosomal RNA precursor (pre-rRNA) biogenesis. We found nucleolar and coiled-body phosphoprotein 1 (NOLC1) as a novel target of FTO. NOLC1 was upregulated after FTO knockdown and promoted DPSC senescence. Mechanistically, FTO downregulation increased the m^6^A modifications of *NOLC1* mRNA, increasing the stability of the *NOLC1* mRNA. NOLC1 upregulation inhibits the transcription of pre-rRNA, causing nucleolar stress and p53 accumulation. In addition, NOLC1 knockdown partially rescued FTO deficiency-induced DPSC senescence. Our findings identified the significant role of the FTO/NOLC1/p53 axis in DPSC senescence and provide new insights to prevent the aging of DPSCs, which is beneficial for the application of DPSCs in regenerative medicine and stem cell therapy.

## 1. Introduction

Dental pulp stem cells (DPSCs), a type of mesenchymal stem cell, can be extensively employed in scientific research and regenerative medicine because of their multidirectional differentiation capacity [1,2,3]. However, their limited proliferation, a result of replicative senescence during in vitro culture, makes large-scale expansion challenging. This limitation poses difficulties for therapeutic applications requiring substantial quantities of DPSCs [2,4]. As DPSCs age, their ability to proliferate, migrate, self-renew, and differentiate declines, which limits their applications [5]. Thus, it is crucial to delve into the mechanisms underlying DPSC senescence and develop counteracting strategies. Several signaling pathways are involved in DPSC senescence. For example, receptor tyrosine kinase-like orphan receptor 2 can inhibit DPSC senescence by enhancing sphingomyelin biogenesis [6]. Melatonin reduces DPSC senescence by downregulating matrix metallopeptidase 3 [7]. Decreased serine metabolism leads to increased p16 expression through reduced DNA methylation, which contributes to the aging of DPSCs [8]. However, the mechanisms underlying cellular senescence in DPSCs are complex and require further investigation.

Messenger RNA (mRNA) frequently undergoes reversible chemical modifications such as N6-methyladenosine (m^6^A), which occurs at the sixth nitrogen atom of adenine. m^6^A plays significant roles in mRNA stability [9] and splicing [10]. It was discovered that the first m^6^A demethylase implicated in the reversible process of m^6^A modification was the fat mass and obesity-associated (FTO) protein [11]. FTO is involved in several physiological and pathological processes. For instance, FTO regulates the alternative splicing of the adipogenesis-related transcription factor runt-related transcription factor 1 by modulating its mRNA m^6^A modification around the splice sites [12]. FTO plays oncogenic roles in several cancers, including acute myeloid leukemia [13] and melanoma [14]. FTO silencing upregulates the stability of the Fos proto-oncogene (*FOS*) mRNA, leading to increased FOS protein expression in granulosa cells, which contributes to ovarian aging [15]. Furthermore, FTO prevents senescence in human mesenchymal progenitor cells by improving the stability of the MIS12 kinetochore complex component (MIS12), which is unrelated to the m^6^A demethylation activity of FTO [16]. In addition, our previous study showed that FTO can promote odontoblastic differentiation in DPSCs by promoting the inclusion of exon 5 of runt-related transcription factor 2 [17]. However, the role of FTO in DPSC senescence remains unclear.

Nucleolar coiled-body phosphorylated protein 1 (NOLC1, also called NOPP140) is a nucleolar protein located in the nucleolar-dense fiber component. NOLC1 dynamically shuttles between the cytoplasm and the nucleus [18,19]. A previous study has demonstrated that NOLC1 overexpression promotes cellular senescence by reducing ribosomal RNA precursor (pre-rRNA) levels [20]. Nevertheless, its function in DPSC senescence is not yet clear, and further research is necessary to ascertain whether m^6^A modifications influence NOLC1 expression.

In this study, we examined the levels of FTO expression in DPSCs during senescence. RNA sequencing was subsequently carried out to identify the genes regulated by FTO to explore the specific role of FTO in DPSC senescence. *NOLC1*, a gene associated with cellular senescence, was determined to be a downstream target of FTO. Next, we used methylated RNA immunoprecipitation (MeRIP)-RT-PCR to detect m^6^A sites on *NOLC1* mRNA and investigated how FTO regulates NOLC1 expression through m^6^A modifications. In summary, our study elucidates the function and underlying mechanisms of FTO in DPSC senescence and provides novel ideas for the prevention of DPSC senescence.

## 2. Materials and Methods

### 2.1. Cell Culture

Primary human DPSCs were cultured as previously described [1]. Briefly, collagenase was used to treat newly extracted dental pulp tissue from pulp cavities. The digested cells were collected and cultured in alpha Minimum Essential Medium (HyClone, Marlborough, MA, USA) containing 20% fetal bovine serum (Gibco, Carlsbad, CA, USA) and 1% antibiotic-antimycotic (Gibco, Carlsbad, CA, USA) in 5% CO_2_ at 37 °C. DPSCs’ surface markers were characterized using antibodies against CD29, CD90, CD146, CD45, and CD34, and analyzed by flow cytometry. The following experiments were conducted using DPSCs at passages 3–12 [6]. The primary human DPSCs were isolated from healthy donors, and informed consent was obtained from all subjects. This study was approved by the Ethics Committee at the Hospital of Stomatology, Wuhan University. Dulbecco’s Modified Eagle’s Medium (HyClone) supplemented with 10% fetal bovine serum and 1% antibiotic-antimycotic solution was used to cultivate human embryonic kidney (HEK) 293 (Cellcook, Guangzhou, China) and HEK 293T cells (Procell, Wuhan, China).

### 2.2. Plasmids

The pCMV3-FTO-Flag plasmid was obtained from Sino Biological Inc. (Beijing, China). And pLVX-FTO-Flag-IRES-ZSgreen1 plasmid was obtained from our laboratory. For overexpression of human NOLC1, NOLC1 cDNA was amplified from DPSCs and a 3 × Flag tag was added to the *NOLC1* coding sequence. The NOLC1 expression plasmid was created by inserting the *NOLC1*-3 × Flag fusion fragment at the SpeI and NotI sites into the pLVX-IRES-Puro (Clontech, Mountain View, CA, USA) vector. To further explore the m^6^A modification of *NOLC1*, the fragments of *NOLC1* open reading frame and 3′ UTR, including wild-type and mutant m^6^A motifs (adenosine was substituted with guanine), were inserted into pEGFP-N1 vector at NheI and BamHI sites. Table S1 lists the primers used to generate plasmids. Lipo293 (Beyotime, Shanghai, China) was used for transfecting HEK 293 cells. HEK 293T cells were used for lentivirus production after transfection with the overexpression plasmids, pMD2.G and, psPAX2 to produce lentivirus in the presence of Lipo293.

### 2.3. RNA Interference

Anti-human *FTO* and *NOLC1* siRNAs were purchased from Sangon Biotech (Shanghai, China). The anti-*FTO* siRNA sequences are 5′-AAAUAGCCGCUGCUUGUGAGA-3′ (siFTO-1) and 5′-GCACAAGCAUGGCUGCUUA-3′ (siFTO-2), respectively. The sequences of siRNAs for *NOLC1* knockdown (KD) are as follows: 5′-CACCAAGAAUUCUUCAAAU-3′ (siNOLC1-1) and 5′-GCGAAAGUUACAGGCAAAU-3′ (siNOLC1-2). Next, siRNA (20 nM) was transfected into DPSCs using Lipofectamine 3000 (Invitrogen, Carlsbad, CA, USA) according to the manufacturer’s instructions.

### 2.4. Quantitative RT-PCR and Semi-Quantitative RT-PCR

An RNA miniprep kit (Axygen, Union City, CA, USA) was utilized to extract total RNA. cDNA was synthesized using Maxima H Minus Reverse Transcriptase (Thermo Fisher Scientific, San Diego, CA, USA). The 2× Taq Master Mix (Vazyme, Nanjing, China) and the ChamQ SYBR qPCR Master Mix (Vazyme, Nanjing, China) were used for PCR. Relative gene expression was normalized to β-actin using the 2^−ΔΔCt^ method. The primer sequences are listed in Table S2.

### 2.5. Western Blot Analysis

After collection using 2× SDS, total protein was separated using a 10% SDS-PAGE gel. Following blotting onto nitrocellulose membranes, the membranes were blocked and incubated with primary antibodies against p53 (Proteintech, Wuhan, China), p16 (ZenBio, Chengdu, China), γH2AX (ZenBio, Chengdu, China), FTO (Proteintech, Wuhan, China), NOLC1 (Abcam, Cambridge, UK), and β-actin (Proteintech).

### 2.6. Reactive Oxygen Species Detection

To detect reactive oxygen species (ROS) produced by DPSCs, a DCFH-DA reactive oxygen species assay kit (Beyotime, Shanghai, China) was utilized. DPSCs were treated with 10 μM DCFH-DA for 20 min at 37 °C in a dark incubator. After washing with PBS and resuspending in staining buffer, a CytoFLEX Flow Cytometer (Beckman Coulter, Brea, CA, USA) was used to quickly measure the mean fluorescence intensity (MFI) of the DPSCs.

### 2.7. β-Galactosidase Staining

To find the senescent DPSCs, a β-galactosidase (β-gal) staining kit (Beyotime, Shanghai, China) was used. Briefly, DPSCs were fixed and stained using the corresponding solutions according to the manufacturer’s instructions. The plates were then placed in a dark incubator. A light microscope was used to count blue-stained senescent DPSCs.

### 2.8. Cell Cycle

After treatment with siRNAs, DPSCs were harvested using a cell cycle detection kit (Multi Sciences, Hangzhou, China) and examined by flow cytometry to assess cell cycle distribution.

### 2.9. RNA Sequencing

DPSCs were transfected with siNC and siFTO using Lipofectamine 3000, and TRIzol reagent (Invitrogen) was used to collect total RNA. Wuhan Ruixing Biotechnology (Wuhan, China) was used for the RNA sequencing. Briefly, the total RNA was purified and reverse-transcribed into cDNA. Paired-end runs were performed for RNA sequencing using a NovaSeq 6000 sequencer (Illumina, San Diego, CA, USA).

### 2.10. MeRIP Assay

The MeRIP assay was performed as previously described [21]. In short, total RNA was extracted and fragmented using an RNA fragmentation buffer containing ZnCl_2_. The m^6^A-antibody was used for m^6^A-immunoprecipitation mixed with fragmented RNA in IP buffer for 4 h. The immunoprecipitated m^6^A-RNAs were captured using Dynabeads Protein A/G (Invitrogen). RNA was then eluted in proteinase K-containing elution buffer. The immunoprecipitated RNA was prepared for subsequent analysis.

### 2.11. RNA Stability Assay

DPSCs with FTO knockdown were treated with actinomycin D (5 μg/mL) and total RNA was extracted after 0, 6, and 12 h. Following reverse transcription, the mRNA levels of the target gene were determined by qPCR. GraphPad Prism 8 software was used to calculate the mRNA half-life.

### 2.12. Statistical Analysis

The comparison of two groups for statistical significance was conducted using Student’s *t*-test (two-tailed). To compare three or more groups, a one-way analysis of variance was utilized and Tukey’s multiple comparison or Dunnett’s multiple comparison test was used to assess the differences between each pair of means. All experiments were independently replicated at least three times (*n* ≥ 3), and “*n*” represents the number of biological replicates. All data were calculated based on biological replicates and are presented as mean ± standard deviation. A *p*-value < 0.05 was considered statistically significant using GraphPad Prism 8 software.

## 3. Results

### 3.1. FTO Expression Levels Were Reduced in Senescent DPSCs

**Figure 1:**
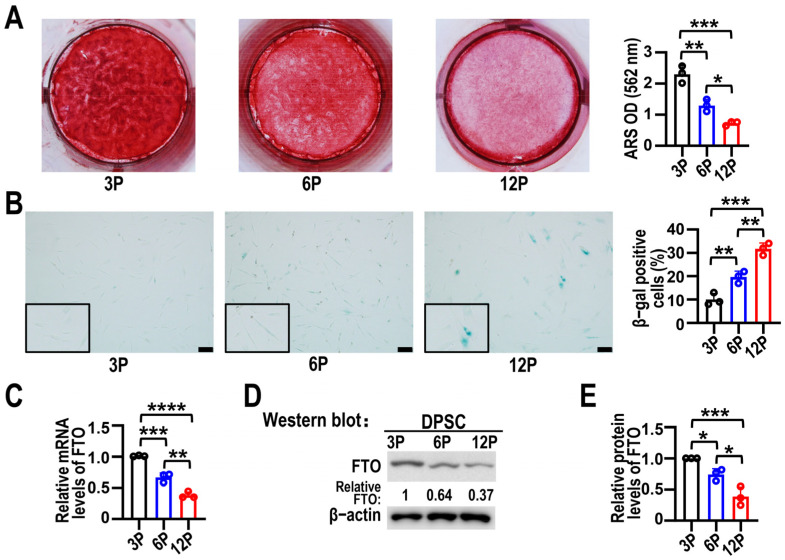
FTO expression was reduced during DPSC senescence. (**A**) Alizarin red S staining of DPSCs at third passage (3P), sixth passage (6P), and twelfth passage (12P) after differentiation induction for 21 days. Relative OD value was summarized by histograms on right. Data are means ± SD, *n* = 3. (**B**) β-gal staining for DPSCs (3P, 6P, and 12P). Scale bars, 100 μm. Histograms represent quantitative analysis of 3P, 6P, and 12P β-gal-stained DPSCs. Data are means ± SD, *n* = 3. (**C**) *FTO* mRNA levels in DPSCs at 3P, 6P, and 12P were analyzed using RT-qPCR. Data are means ± SD, *n* = 3. (**D**) Western blotting of FTO protein expression levels of DPSCs at 3P, 6P, and 12P. β-actin served as loading control. The original Western blot figure is in the Supplementary Materials, Figure S2. (**E**) Analysis of FTO protein expression in DPSCs at 3P, 6P, and 12P statistically shown in histograms. Data are means ± SD. In this figure, statistical analysis was performed by one-way ANOVA, *n* = 3. * *p* < 0.05, ** *p* < 0.01, *** *p* < 0.001 and **** *p* < 0.0001.

The DPSCs used in this study expressed CD29, CD90, and CD146, but not CD34 or CD45, confirming their mesenchymal stem cell identity (Figure S1). Previous research has indicated that DPSCs cultivated at passage six could exhibit a senescence phenotype [22]. Alizarin red S staining revealed that, compared to the third passage, the capacity of the sixth and twelfth passages of DPSCs to form mineralized nodules was gradually reduced (Figure 1A). Senescence was further corroborated by increased β-gal activity, which was modestly elevated at passage six and markedly higher by passage twelve (Figure 1B). We then assessed FTO expression levels during DPSC senescence. RT-qPCR and Western blot analyses revealed that as the passage number of DPSCs increased, the expression of FTO decreased noticeably (Figure 1C–E). These results indicated that FTO expression was decreased in senescent DPSCs.

### 3.2. Depletion of FTO Accelerated DPSC Senescence

**Figure 2:**
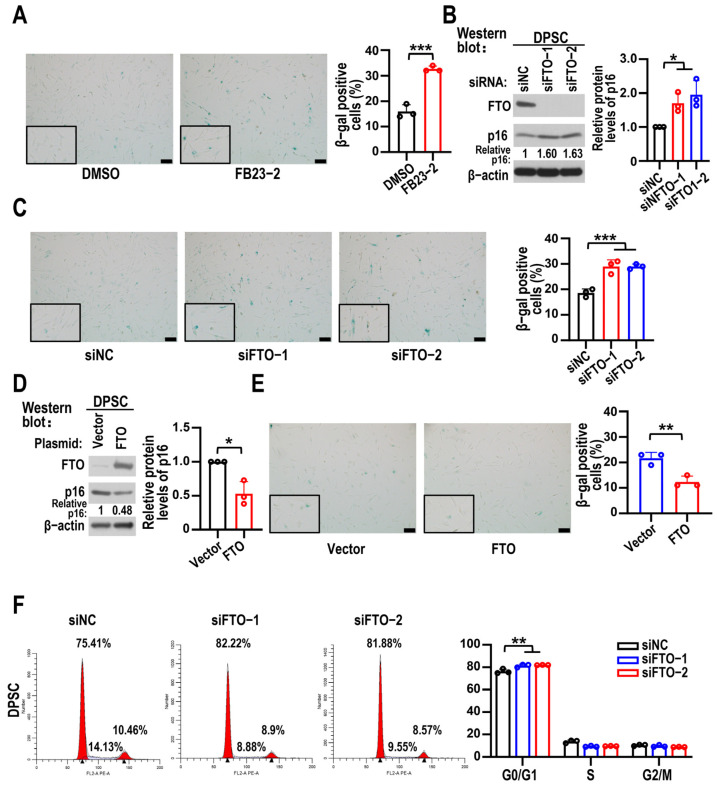
FTO counteracted DPSC senescence. (**A**) β-gal staining for DPSCs at sixth passage (6P) exposed to 5 μM FB23-2. Scale bars, 100 μm. β-gal-positive DPSC statistical results shown in the diagram on right. Data are means ± SD, and were analyzed by two-tailed Student’s *t*-test, *n* = 3. (**B**) The effects of FTO knockdown on p16 protein levels in DPSCs were analyzed by Western blot analysis. β-actin served as loading control. Data are means ± SD, and were analyzed by one-way ANOVA, *n* = 3. The original Western blot figure is in the Supplementary Materials, Figure S3. (**C**) β-gal staining for DPSCs at 6P transfected with siFTO (siFTO-1 and siFTO-2) or control siRNA (siNC). Scale bars, 100 μm. The histogram on right shows statistical analysis of β-gal-positive DPSCs. Data are means ± SD, and were analyzed by one-way ANOVA, *n* = 3. (**D**) The effects of FTO overexpression on p16 protein levels in DPSCs at 6P were analyzed by Western blot analysis. Data are means ± SD, and were analyzed by two-tailed Student’s *t*-test, *n* = 3. The original Western blot figure is in the Supplementary Materials, Figure S4. (**E**) β-gal staining of overexpression vector (control) or FTO in DPSCs at 6P. The diagram on right displays β-gal positive DPSC statistical results. Scale bars, 100 μm. Data are means ± SD, and were analyzed by two-tailed Student’s *t*-test, *n* = 3. (**F**) Cell cycle distribution was detected by flow cytometry in cells at 3P transfected with siFTO (siFTO-1 and siFTO-2) or control siRNA (siNC). Histograms show statistical analysis results of cell cycle distribution. Data are means ± SD, and were analyzed by one-way ANOVA, *n* = 3. * *p* < 0.05, ** *p* < 0.05 and *** *p* < 0.001.

To elucidate the functional role of FTO in DPSC senescence, gain- and loss-of-function experiments were performed. We used β-gal staining to represent the extent of cellular senescence. Compared to the DMSO group, DPSCs treated with the FTO inhibitor FB23-2 exhibited greater levels of β-gal activity (Figure 2A). The protein level of p16 increased following FTO knockdown, and more senescent cells were detected after treatment of DPSCs with FTO KD (Figure 2B,C). Overexpression of FTO reduced p16 protein levels and β-gal-positive cells significantly (Figure 2D,E). To examine the biological function of FTO in the cell cycle distribution of DPSCs, flow cytometric analysis was carried out. FTO KD resulted in a cell cycle arrest at G0/G1 phase in DPSCs (Figure 2F).

**Figure 3:**
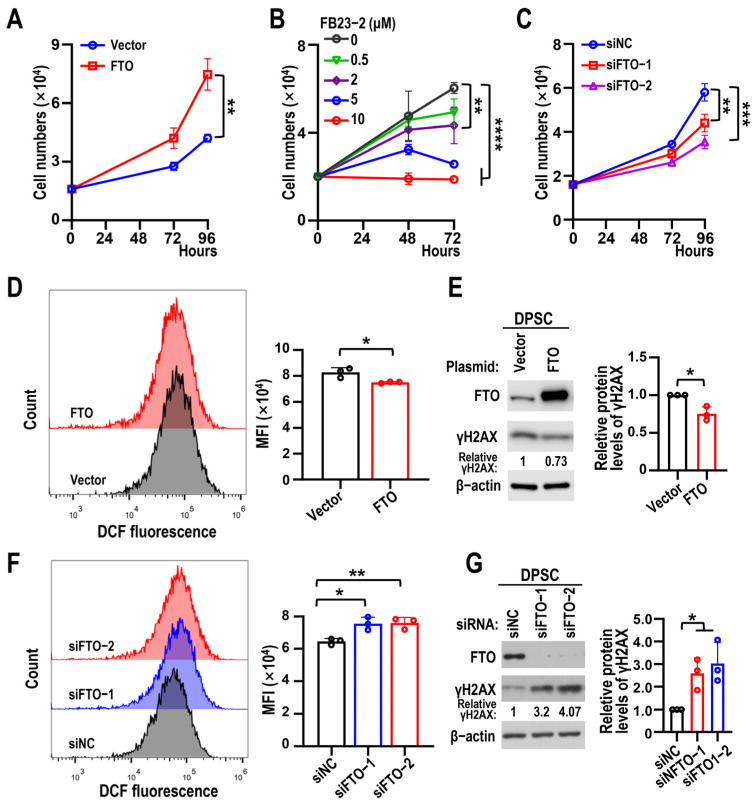
FTO promotes DPSC proliferation and decreases ROS accumulation. (**A**) Proliferation curves of DPSCs at sixth passage (6P) with overexpression of FTO and vector (control). Data are means ± SD, and were analyzed by two-tailed Student’s *t*-test, *n* = 3. (**B**) Proliferation curves of DPSCs at 6P treated with 0, 0.5, 2, 5, and 10 μM of FB23-2. Data are means ± SD, and were analyzed by one-way ANOVA, *n* = 3. (**C**) Proliferation curves of DPSCs at 6P transfected with FTO siRNAs (siFTO-1 and siFTO-2) or control siRNA (siNC). Data are means ± SD, and were analyzed by one-way ANOVA, *n* = 3. (**D**) DPSCs at 6P transfected with FTO or vector control plasmids. ROS levels were measured using flow cytometry. Histogram on right shows mean fluorescence intensity (MFI). Data are means ± SD, and were analyzed by two-tailed Student’s *t*-test, *n* = 3. (**E**) The effects of FTO overexpression on γH2AX protein levels in DPSCs were analyzed by Western blot analysis. Relative protein levels of γH2AX normalized to β-actin. β-actin served as loading control. Data are means ± SD, and were analyzed by two-tailed Student’s *t*-test, *n* = 3. The original Western blot figure is in the Supplementary Materials, Figure S5. (**F**) Flow cytometry analysis of ROS after DPSCs at 3P transfected with siFTO-1 and siFTO-2. siNC served as control. Data are means ± SD, and were analyzed by one-way ANOVA, *n* = 3. (**G**) Effects of FTO knockdown on γH2AX protein levels in DPSCs were analyzed by Western blot analysis. Data are means ± SD, and were analyzed by one-way ANOVA, *n* = 3. The original Western blot figure is in the Supplementary Materials, Figure S6. * *p* < 0.05, ** *p* < 0.01, *** *p* < 0.001 and **** *p* < 0.0001.

In addition, the proliferation rate of DPSCs increased after FTO overexpression (Figure 3A) but decreased significantly in a dose-dependent manner upon FB23-2 treatment (Figure 3B). FTO KD inhibited DPSC proliferation (Figure 3C). Since the accumulation of ROS can cause DNA damage and lead to cellular senescence [23], we examined ROS levels and γH2AX expression in DPSCs following FTO overexpression or KD. These findings showed that ROS and protein levels of γH2AX decreased after FTO overexpression (Figure 3D,E) and increased with FTO KD (Figure 3F,G). Collectively, these results indicate that FTO depletion accelerates DPSC senescence.

### 3.3. FTO KD Suppresses Pre-rRNA Synthesis Through NOLC1

**Figure 4:**
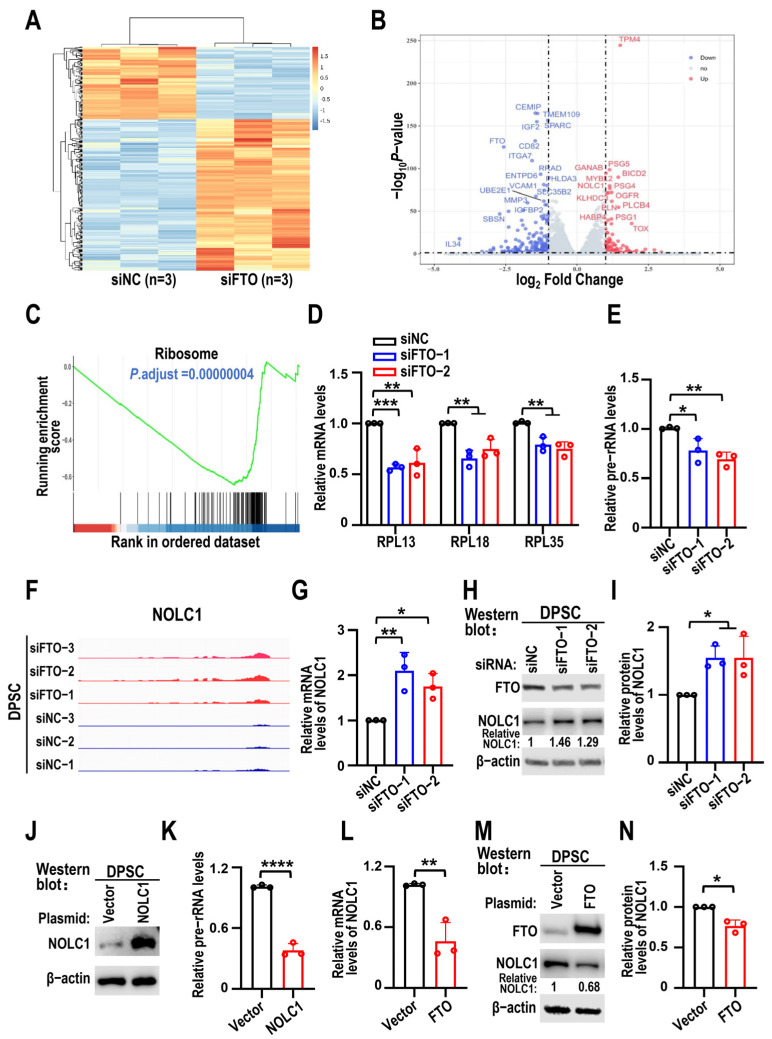
Knockdown of FTO decreases pre-rRNA synthesis and promotes NOLC1 expression in DPSCs. (**A**) Heat map displays siFTO and siNC genes differentially expressed in DPSCs. (**B**) The volcano map depicts the differentially expressed genes between the two groups, with elevated genes represented by red dots and downregulated genes by blue dots. (**C**) Ribosome pathway depicted by GSEA. (**D**) RT-qPCR results of *RPL13*, *RPL18*, and *RPL35* after FTO KD. Data are means ± SD, and were analyzed by one-way ANOVA, *n* = 3. (**E**) Pre-rRNA levels analyzed using RT-qPCR after transfection with FTO siRNAs (siFTO-1 and siFTO-2) in DPSCs. siNC was used as control. Data are means ± SD, and were analyzed by one-way ANOVA, *n* = 3. (**F**) *NOLC1* mRNA expression levels after FTO knockdown visualized via IGV software (version 2.12.2). (**G**) DPSCs transfected with FTO siRNAs (siFTO-1 and siFTO-2) or siNC. *NOLC1* mRNA levels analyzed using RT-qPCR. Data are means ± SD, and were analyzed by one-way ANOVA, *n* = 3. (**H**,**I**) The effects of FTO knockdown on NOLC1 protein levels in DPSCs were analyzed by Western blot analysis. Relative protein levels of NOLC1 normalized to β-actin. β-actin served as loading control. Data are means ± SD, and were analyzed by one-way ANOVA, *n* = 3. The original Western blot figure is in the Supplementary Materials, Figure S7. (**J**) NOLC1 overexpression in DPSCs was verified by Western blot. The original Western blot figure is in the Supplementary Materials, Figure S8. (**K**) RT-qPCR performed to evaluate pre-rRNA levels following NOLC1 overexpression. Data are means ± SD, and were analyzed by two-tailed Student’s *t*-test, *n* = 3. (**L**) RT-qPCR performed to evaluate *NOLC1* mRNA levels following FTO overexpression. Data are means ± SD, and were analyzed by two-tailed Student’s *t*-test, *n* = 3. (**M**,**N**) The effects of FTO overexpression on NOLC1 protein levels in DPSCs were analyzed by Western blot analysis. Relative protein levels of NOLC1 normalized to β-actin. Data are means ± SD, and were analyzed by two-tailed Student’s *t*-test, *n* = 3. The original Western blot figure is in the Supplementary Materials, Figure S9. * *p* < 0.05, ** *p* < 0.01, *** *p* < 0.001 and **** *p* < 0.0001.

We performed RNA sequencing of the FTO KD and control groups to screen the downstream target genes of FTO in DPSC senescence and found 341 differentially expressed genes (DEGs). Among these, 111 were upregulated and 230 were downregulated, according to the criteria of corrected *p*-value < 0.05 and |log2FC| > 1 (Figure 4A). These DEGs were visualized in a volcano plot with genes showing significant fold changes (Figure 4B). Gene set enrichment analysis (GSEA) indicated that the ribosomal pathway was the most significantly altered (Figure 4C). Following FTO KD, many genes encoding ribosomal proteins were downregulated, leading to impaired ribosome synthesis. We selected three anti-aging ribosomal protein-coding genes for validation [24,25]. RT-qPCR results showed RPL13, RPL18, and RPL35 were downregulated after FTO KD (Figure 4D). Subsequently, the transcript levels of pre-rRNA significantly decreased after FTO silencing, as shown by RT-qPCR (Figure 4E). Among these DEGs, NOLC1 showed promise because of its involvement in rRNA synthesis and senescence. The IGV views indicated that NOLC1 expression was upregulated after FTO KD (Figure 4F). To confirm that NOLC1 is a downstream target gene of FTO, the upregulation of NOLC1 mRNA and protein levels after FTO silencing was confirmed by RT-qPCR and Western blot (Figure 4G–I). Consistent with previous findings [20], NOLC1 overexpression downregulated the expression of pre-rRNA (Figure 4J,K). Moreover, FTO overexpression decreased NOLC1 mRNA and protein levels (Figure 4L–N).

### 3.4. NOLC1 Promotes DPSC Senescence

**Figure 5:**
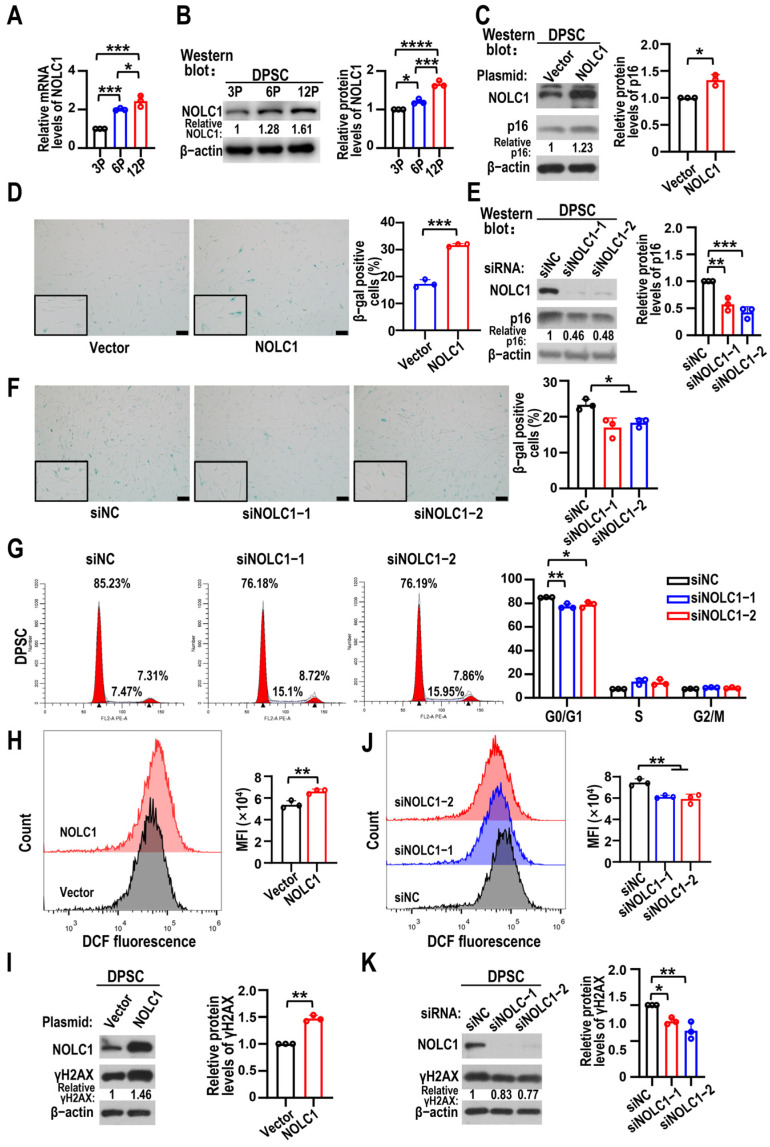
NOLC1 promotes DPSC senescence and ROS accumulation. (**A**) *NOLC1* mRNA levels in 3P, 6P, and 12P DPSCs determined using RT-qPCR. Data are means ± SD, and were analyzed by one-way ANOVA, *n* = 3. (**B**) NOLC1 protein expression levels of DPSCs at 3P, 6P, and 12P were detected by Western blot. Relative NOLC1 protein expression levels were summarized in histogram on right. Data are means ± SD, and were analyzed by one-way ANOVA, *n* = 3. The original Western blot figure is in the Supplementary Materials, Figure S10. (**C**) The effects of NOLC1 overexpression on p16 protein levels in DPSCs were analyzed by Western blot analysis. β-actin served as loading control. Data are means ± SD, and were analyzed by two-tailed Student’s *t*-test, *n* = 3. The original Western blot figure is in the Supplementary Materials, Figure S11. (**D**) DPSCs at 6P overexpressing vector (control) or NOLC1 detected by β-gal staining. Scale bars, 100 μm. The histogram on right shows statistical analysis of β-gal-positive DPSCs. Data are means ± SD, and were analyzed by two-tailed Student’s *t*-test, *n* = 3. (**E**) The effects of NOLC1 knockdown on p16 protein levels in DPSCs shown by Western blot analysis. Data are means ± SD, and were analyzed by one-way ANOVA, *n* = 3. The original Western blot figure is in the Supplementary Materials, Figure S12. (**F**) β-gal staining of DPSCs at 6P transfected with siNOLC1 (siNOLC1-1 and siNOLC1-2) or control siRNA (siNC). Scale bars, 100 μm. The histogram on right shows statistical analysis of β-gal-positive DPSCs. Data are means ± SD, and were analyzed by one-way ANOVA, *n* = 3. (**G**) Cell cycle distribution was detected by flow cytometry in cells transfected with siNOLC1 (siNOLC1-1 and siNOLC1-2) or control siRNA (siNC). Histograms show statistical analysis results of cell cycle distribution. Data are means ± SD, and were analyzed by one-way ANOVA, *n* = 3. (**H**) DPSCs transfected with NOLC1 or vector (control). Flow cytometry used to measure ROS levels. Histogram displays mean fluorescence intensity (MFI) on the right. Data are means ± SD, and were analyzed by two-tailed Student’s *t*-test, n = 3. (**I**) The effects of NOLC1 overexpression on γH2AX protein levels in DPSCs was analyzed by Western blot analysis. Relative protein levels of γH2AX normalized to β-actin. Data are means ± SD, and were analyzed by two-tailed Student’s *t*-test, *n* = 3. The original Western blot figure is in the Supplementary Materials, Figure S13. (**J**) Flow cytometry analysis of ROS in DPSCs transfected with siNOLC1-1 or siNOLC1-2. siNC used as control. Data are means ± SD, and were analyzed by one-way ANOVA, *n* = 3. (**K**) The effects of NOLC1 knockdown on γH2AX protein levels in DPSCs was analyzed by Western blot analysis. Relative protein levels of γH2AX normalized to β-actin. Data are means ± SD, and were analyzed by one-way ANOVA, *n* = 3. The original Western blot figure is in the Supplementary Materials, Figure S14. * *p* < 0.05, ** *p* < 0.01, *** *p* < 0.001 and **** *p* < 0.0001.

Next, we detected the expression levels of NOLC1 during DPSC senescence. As expected, both the transcriptional and protein levels of NOLC1 increased with DPSC senescence (Figure 5A,B), suggesting its positive role in DPSC senescence. Moreover, the protein levels of p16 and β-gal-positive DPSCs increased as a result of overexpressing NOLC1 (Figure 5C,D), whereas the number of senescent cells and p16 protein levels reduced after NOLC1 KD (Figure 5E,F). Moreover, NOLC1 KD resulted in a decrease in the proportion of DPSCs at the G0/G1 phase (Figure 5G). ROS and the protein levels of γH2AX increased following NOLC1 overexpression (Figure 5H,I) and decreased after NOLC1 KD (Figure 5J,K). These results confirmed that NOLC1 could promote DPSC senescence.

**Figure 6:**
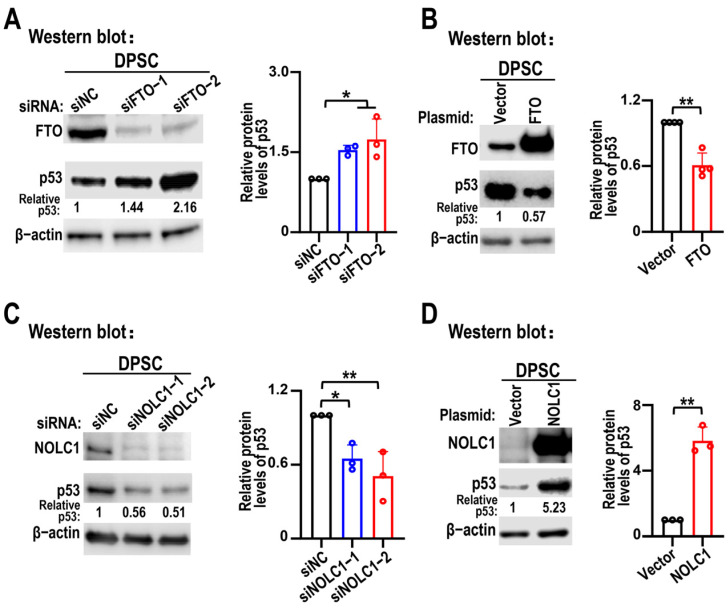
FTO and NOLC1 regulate p53 protein levels. (**A**,**B**) Effects of FTO knockdown and overexpression on p53 protein levels in DPSCs were analyzed by Western blot analysis; siNC and vector were used as negative controls, respectively. Data are presented as means ± SD; FTO KD data (*n* = 3) were analyzed by one-way ANOVA, and FTO overexpression data (*n* = 4) were analyzed by a two-tailed Student’s *t*-test. The original Western blot figures are in the Supplementary Materials, Figures S15 and S16. (**C**,**D**) Effects of NOLC1 silencing and overexpression on p53 protein levels in DPSCs as shown by Western blot; siNC and vector were used as negative controls, respectively. Relative p53 protein levels were normalized to β-actin. Histograms show statistical analysis results of relative p53 protein levels. Data are means ± SD, *n* = 3, and were analyzed by one way ANOVA for NOLC1 KD, two-tailed Student’s *t*-test for NOLC1 overexpression. The original Western blot figures are in the Supplementary Materials, Figures S17 and S18. * *p* < 0.05, ** *p* < 0.01.

Given that p53 is associated with nucleolar stress and is a hallmark of senescence, p53 protein levels were analyzed following FTO overexpression and KD. Western blot analysis showed that p53 protein levels were upregulated after FTO KD in DPSCs (Figure 6A), whereas FTO overexpression decreased p53 protein levels (Figure 6B). Next, we examined the p53 protein levels after NOLC1 KD and overexpression. Western blotting showed a positive correlation between NOLC1 and p53 protein expression levels (Figure 6C,D). This suggests that FTO depletion induces DPSC senescence by increasing NOLC1 expression.

### 3.5. NOLC1 Is Regulated by an FTO-m^6^A-Dependent Mechanism, and NOLC1 KD Partially Rescues FTO Silencing-Induced DPSC Senescence

**Figure 7:**
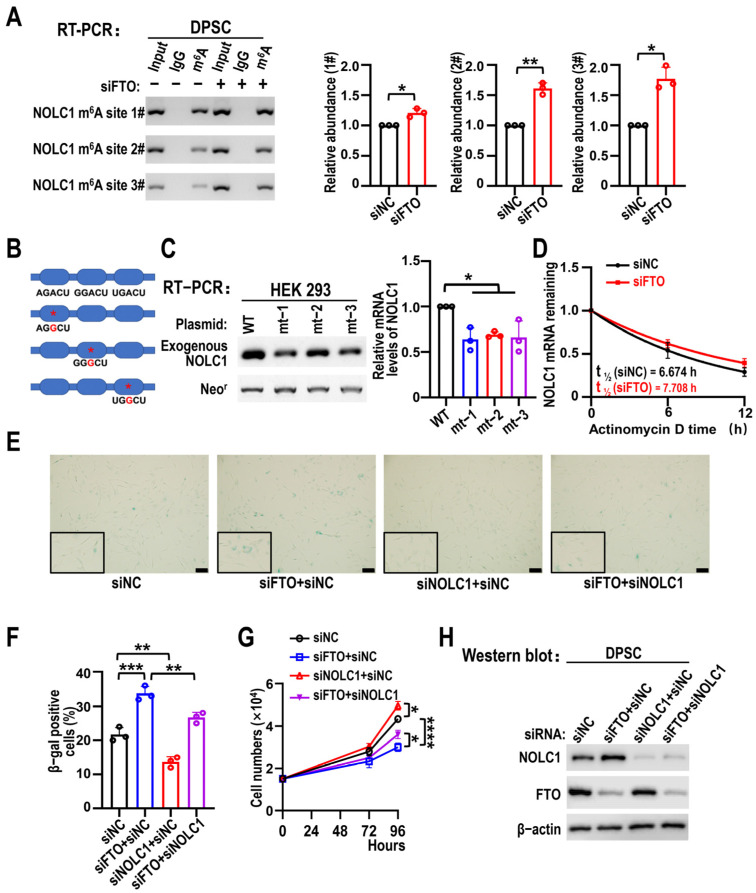
NOLC1 is regulated by an FTO-m^6^A-dependent mechanism, and NOLC1 knockdown partly rescues DPSC senescence induced by FTO silencing. (**A**) Effects of FTO knockdown on m^6^A-modification of *NOLC1* mRNA in DPSCs after MeRIP. RT-PCR used to measure mRNA levels. Relative mRNA abundance analyzed by input sample. Data are means ± SD, and were analyzed by two-tailed Student’s *t*-test, *n* = 3. The original RT-PCR figure is in the Supplementary Materials, Figure S20. (**B**) m^6^A sequence mutant or wild-type NOLC1 inserted into pEGFP-N1 vector. m^6^A site mutations created by substituting adenosine with guanine. (**C**) RT-PCR results of transcription levels of exogenous NOLC1 transfected with NOLC1 wild-type or three mutant m^6^A site plasmids in HEK 293 cells. Neomycin resistance gene (Neo^r^) served as a loading control. Data are means ± SD, and were analyzed by one-way ANOVA, *n* = 3. The original RT-PCR figure is in the Supplementary Materials, Figure S21. (**D**) RT-qPCR analysis of *NOLC1* mRNA half-life in DPSCs after transfection with siNC and siFTO. *n* = 3. (**E**) DPSCs transfected with FTO or NOLC1 siRNAs stained to assess β-gal activity. Transfected cells divided into four groups: siNC, siFTO + siNC, siNOLC1 + siNC, and siFTO + siNOLC1. siRNA (40 nM) was transfected into DPSCs. Scale bars, 100 μm. (**F**) Statistical analysis of β-gal-positive cells of siNC, siFTO + siNC, siNOLC1 + siNC, and siFTO + siNOLC1. Data are means ± SD, and were analyzed by one-way ANOVA, *n* = 3. (**G**) Proliferation curves of DPSCs treated with FTO or NOLC1 siRNAs. Four groups calculated after transfection: siNC, siFTO + siNC, siNOLC1 + siNC, and siFTO + siNOLC1. siRNA (40 nM) was transfected into DPSCs. Data are means ± SD, and were analyzed by one-way ANOVA, *n* = 3. (**H**) Western blotting revealed efficacy of FTO knockdown or NOLC1 knockdown in DPSCs. The original Western blot figure is in the Supplementary Materials, Figure S19. * *p* < 0.05, ** *p* < 0.01, *** *p* < 0.001 and **** *p* < 0.0001.

To clarify whether FTO regulates the expression of NOLC1 through m^6^A modification, we first predicted m^6^A sites on *NOLC1* mRNA using an online tool and then conducted MeRIP-RT-PCR. This analysis identified three high-confidence m^6^A sites in *NOLC1* mRNA. As expected, RT-PCR following MeRIP with an m^6^A-specific antibody revealed increased enrichment of *NOLC1* mRNA in FTO-silenced DPSCs compared to controls. Three potential m^6^A sites were identified in the *NOLC1* mRNA (Figure 7A). Next, we cloned the entire open reading frame and 3′ UTR of *NOLC1* containing these m^6^A sites into pEGFP-N1 and created m^6^A mutant plasmids of *NOLC1* by substituting guanine for the adenosine at each m^6^A site (Figure 7B). RT-PCR results showed that exogenous *NOLC1* transcription levels were reduced following m^6^A site mutations compared to those in the wild-type control (Figure 7C). In addition, RT-qPCR results showed that *NOLC1* mRNA stability was increased after FTO KD in the actinomycin D experiment (Figure 7D). Next, we observed that NOLC1 downregulation partially rescued FTO KD-induced DPSC senescence. NOLC1 KD alleviated the senescence of DPSCs caused by FTO KD (Figure 7E,F). The decreased proliferative capacity of DPSCs caused by FTO deficiency was partly restored by NOLC1 KD (Figure 7G). Western blot analysis confirmed the successful downregulation of both FTO and NOLC1 at the protein level (Figure 7H). These results demonstrate that FTO silencing promotes NOLC1 expression through an m^6^A-dependent mechanism.

## 4. Discussion

As an m^6^A demethylase, FTO plays essential roles in several physiological and pathological processes in stem cells. According to Wei et al., FTO knockout resulted in closed chromatin in mouse embryonic stem cells, leading to decreased proliferation and defective differentiation capacity of mouse embryonic stem cells [26]. Furthermore, our earlier study demonstrates that FTO promotes DPSC differentiation into odontoblastic cells [17]. These findings underscore the close relationship between FTO and stem cell fate, highlighting the need for further exploration of FTO function in DPSCs. In this study, we discovered that downregulation of FTO accelerated DPSC senescence. Furthermore, RNA sequencing identified *NOLC1* as a novel target gene of FTO in DPSC senescence, revealing that FTO KD decreased pre-rRNA levels. Our findings suggest a novel regulatory axis of FTO/NOLC1/p53 in DPSC senescence, primarily through the inhibition of pre-rRNA synthesis.

Stem cell senescence is an intricate phenomenon that can hinder tissue regeneration, potentially leading to organ failure and increased mortality [27]. To better understand the senescence process and find possible targets for anti-aging, investigating the molecular mechanisms underlying stem cell senescence is of great significance. In regenerative medicine, DPSCs are considered an optimal cell source because of their pluripotency and high proliferation rates [28]. DPSCs can undergo senescence after passaging in vitro, which poses significant challenges to their clinical use [29]. Senescent DPSCs show reduced capabilities in self-renewal, migration, and osteogenic differentiation, thereby limiting their function in dental repair and regeneration [30]. A recent study indicated that senescent DPSCs generated through cell passaging had considerably higher senescence indices at the sixth passage than at the third passage [22]. Thus, the mechanisms underlying senescence were examined using passages three, six, and twelve. In this study, FTO expression decreased with replicative senescence and that FTO KD accelerated the senescence of DPSCs, suggesting that FTO may help delay the senescence of DPSCs. Additionally, FTO plays essential roles in cellular senescence via many signaling pathways. FTO reduces heterochromatin loss in an m^6^A-dependent manner by stabilizing lysine acetyltransferase 8, thereby counteracting skin aging [31]. FTO KD is associated with a senescent phenotype in mouse myoblasts [32]. In addition, FTO counteracts senescence in embryonic stem cells by maintaining MIS12 in an m^6^A-independent manner [16]. Collectively, our research, along with existing studies, demonstrated the essential role of FTO in cell senescence, offering promising prospects for clinical applications in regenerative medicine.

Nucleolar stress caused by disrupted rRNA synthesis can trigger cell senescence [20]. Errors in ribosomal biogenesis lead to alterations in the function and shape of the nucleolus, a phenomenon known as nucleolar stress, which is often associated with p53 accumulation [33]. However, the relationship between FTO and nucleolar stress remains unclear. Here, RNA sequencing results of FTO KD highlighted that the ribosome pathway was most significantly affected in the GSEA, and many ribosomal proteins were downregulated. The reduction in ribosomal protein L35, ribosomal protein L18, and ribosomal protein L13 could induce cellular senescence, according to previous studies [24,25]. Moreover, reduced ribosomal protein levels indicated anomalies in ribosomal biosynthesis, including precursor rRNA transcription and processing [34,35]. In this study, we found that FTO KD notably reduced the transcription levels of pre-rRNA. Similarly, a previous study also showed that in B-cell acute lymphoblastic leukemia cells, FTO inhibition caused nucleolar stress by suppressing pre-rRNA levels [36]. Additionally, p53 protein levels significantly increased following FTO KD. Collectively, these results demonstrate that pre-rRNA transcriptional inhibition caused by FTO KD may result in nucleolar stress and p53 protein accumulation, ultimately leading to DPSC senescence.

Previous studies have identified various factors and signaling pathways that can trigger nucleolar stress [37,38]. Here, we identified *NOLC1* as an FTO downstream target gene through analyzing RNA-seq data of DPSCs because of its function in rRNA synthesis. We found that NOLC1 overexpression significantly reduced the transcript levels of pre-rRNA levels, which were in accordance with a previous study [20]. Surprisingly, our study revealed that NOLC1 was regulated by FTO in an m^6^A mechanism. FTO KD prolongs the half-life of NOLC1 mRNA, indicating that m^6^A modification increases *NOLC1* mRNA stability. However, whether the m^6^A modification affects *NOLC1* mRNA translation remains unclear. In addition, m^6^A reader proteins also play significant roles in controlling the fate of target transcripts. For instance, in mesenchymal stem cell (MSC), knockdown of AlkB homolog 5 RNA demethylase (ALKBH5, another m^6^A eraser) induced senescence by upregulating the expression of cytochrome P450 family 1 subfamily B member 1 (CYP1B1) and causing mitochondrial dysfunction. The m^6^A sites on *CYP1B1* mRNA are recognized by the m^6^A reader insulin like growth factor 2 mRNA binding protein 1 (IGF2BP1), which stabilizes *CYP1B1* mRNA [39]. The m^6^A readers contributing to NOLC1 regulation or DPSC senescence require further investigation in future research. Furthermore, our results demonstrated that NOLC1 overexpression promoted the senescence of DPSCs, with NOLC1 levels increasing during replicative senescence. We also discovered a positive relationship between NOLC1 and p53 protein levels, consistent with the results of a previous study [40]. Notably, NOLC1 KD partially rescued the proliferative and senescent phenotypes of DPSCs induced by FTO deficiency.

We identified three m^6^A modification sites in *NOLC1* mRNA in HEK 293 cells. However, m^6^A-dependent regulation can be cell-type specific. These m^6^A modification sites of *NOLC1* mRNA may require further validation in DPSC. It is a limitation in this study that could be addressed in future research. NOLC1 is a multi-functional protein and involved in several cellular processes such as transcription [41] and protein transport [42]. Our results demonstrated that NOLC1 overexpression decreased rRNA levels and leading to cellular senescence. However, theoretically, NOCL1 may also regulate senescence via other pathways. More research is required to address this limitation.

## 5. Conclusions

Our study concluded that FTO downregulation may accelerate DPSC senescence through NOLC1 by inhibiting rRNA synthesis. These findings suggest that the FTO/NOLC1/p53 axis may serve as an optimal target for counteracting senescence in DPSCs.

## Data Availability

The RNA sequencing raw data have been uploaded to the Genome Sequence Archive for Human (https://ngdc.cncb.ac.cn/gsa-human (accessed on 9 October 2024)) with the accession number HRA008866.

## Supplementary Materials

The following supporting information can be downloaded at: https://www.mdpi.com/article/10.3390/biom15111627/s1, Figure S1: Characterization of DPSCs; Figures S2–S21: Original Western blot and RT-PCR images; Table S1: Primers used for plasmid construction; Table S2: Primers used in RT-PCR and RT-qPCR.
