# Supplementary material for: FTO Suppresses Dental Pulp Stem Cell Senescence by Destabilizing NOLC1 mRNA
Source: Biomolecules. 2025 Nov 19;15(11):1627. doi: 10.3390/biom15111627 (PMC12650298; doi:10.3390/biom15111627)
Supplement: Supplementary file 1 [file biomolecules-15-01627-s001.zip › Supplementary Figure S1 and Table S1 and S2.pdf]

Supplementary Materials

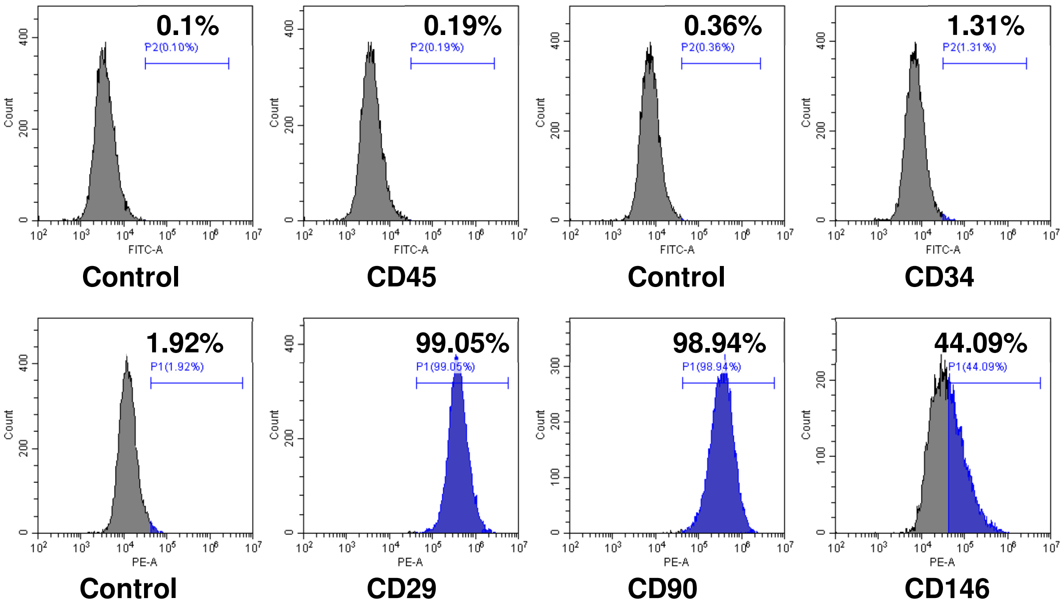

Figure S1. Characterization of DPSCs. Surface protein profiles of DPSCs were analyzed by flow cytometry. DPSCs were negative for the hemopoietic stem cell markers CD45 and CD34 but expressed mesenchymal stem cell markers such as CD29, CD90, and CD146.

Table S1. Primers used for plasmid construction.

	Forward	Reverse
NOLC1 cDNA	TTCCGACTAGTATGGCGGACGCCGGCATTCG	TTCCGGGATCCCTCGCTGTCAAACTTAATAGAATTG
NOLC1-3′UTR	TTCCGGCTAGCATGGCGGACGCCGGCATTCG	TTCCGGGATCCCACATTATAAACCAGCCAG-TTTATTAT
mt1	TAACTTCTGTCAGGGCAGAGGCTGTACCTAAAAA-GCAAGTT	AACTT-GCTTTTTAGGTACAGCCTCTGCCCTGACAGAAGTTA
mt2	ATTGAGTTCTAAGTTGAGGGCTG-CATCTTCTCGTTTTTTAC	GTAAAAAACGAGAAGATGCAGCCCTCAACTTA-GAACTCAAT
mt3	GGTATGGCTGCAGGAAGTGGCTGAGCAGTAGCGG-TACTCAG	CTGAGTACCGCTACTGCTCAGCCACTTCCTG-CAGCCATACC

Table S2. Primers used in RT-PCR and RT-qPCR.

Gene	Forward	Reverse
GAPDH	GAAGGTGAAGGTCGGAGTC	GAAGATGGTGATGGGATTTC
RPL18	ACCAACTCCACATTCAACCAG	TCAGTTTGGGTACCTCCTGAA
RPL13	AAGAAGGGAGACAGTTCTGCTG	GTGATGACTCGAGCTTTCTCCT
RPL35	GCTCTCTAAGATCCGAGTCGTC	CTTGCCCTTGTAGAATTTCCTG
FTO	CCCGAACATTACCTGCTGAT	TTCCAGAAGCTGACCTCTGAG
NOLC1	TTCCTGCGCGATAACCAACTC	CCTGTAACTTTCGCTCTGGGA
pre-rRNA	GCGGAGGTTTAAAGACCC	CCAGACGAGACAGCAAAC
β-actin	CCCAGCACAATGAAGATCAA	ACATCTGCTGGAAGGTGGAC
NOLC1 m^6A *site 1#*	ACCATAACTTCTGTCAGGGCA	GGGACTGAACTGTAGGGACC
NOLC1 m^6A *site 2#*	TGACAACTTGACCCTTCCTAGT	ATCACCACTGCAACCAAGAC
NOLC1 m^6A *site 3#*	ACCTGGGAGCAATTGACATG	ACTCTTCCCTCTCCGTCTTG
exogenous NOLC1	ATGTACTGTTCATGCTGACACA	GCTCCTCGCCCTTGCTCACCA
Neo^r	ATTGAACAAGATGGATTGCACGC	TCAAGAAGGCGATAGAAGGCG
