# Supplementary material for: FTO Suppresses Dental Pulp Stem Cell Senescence by Destabilizing NOLC1 mRNA
Source: Biomolecules. 2025 Nov 19;15(11):1627. doi: 10.3390/biom15111627 (PMC12650298; doi:10.3390/biom15111627)
Supplement: Supplementary file 1 [file biomolecules-15-01627-s001.zip › Supplementary Figure S2-S21 (Original western blot and RT-PCR images).pdf]

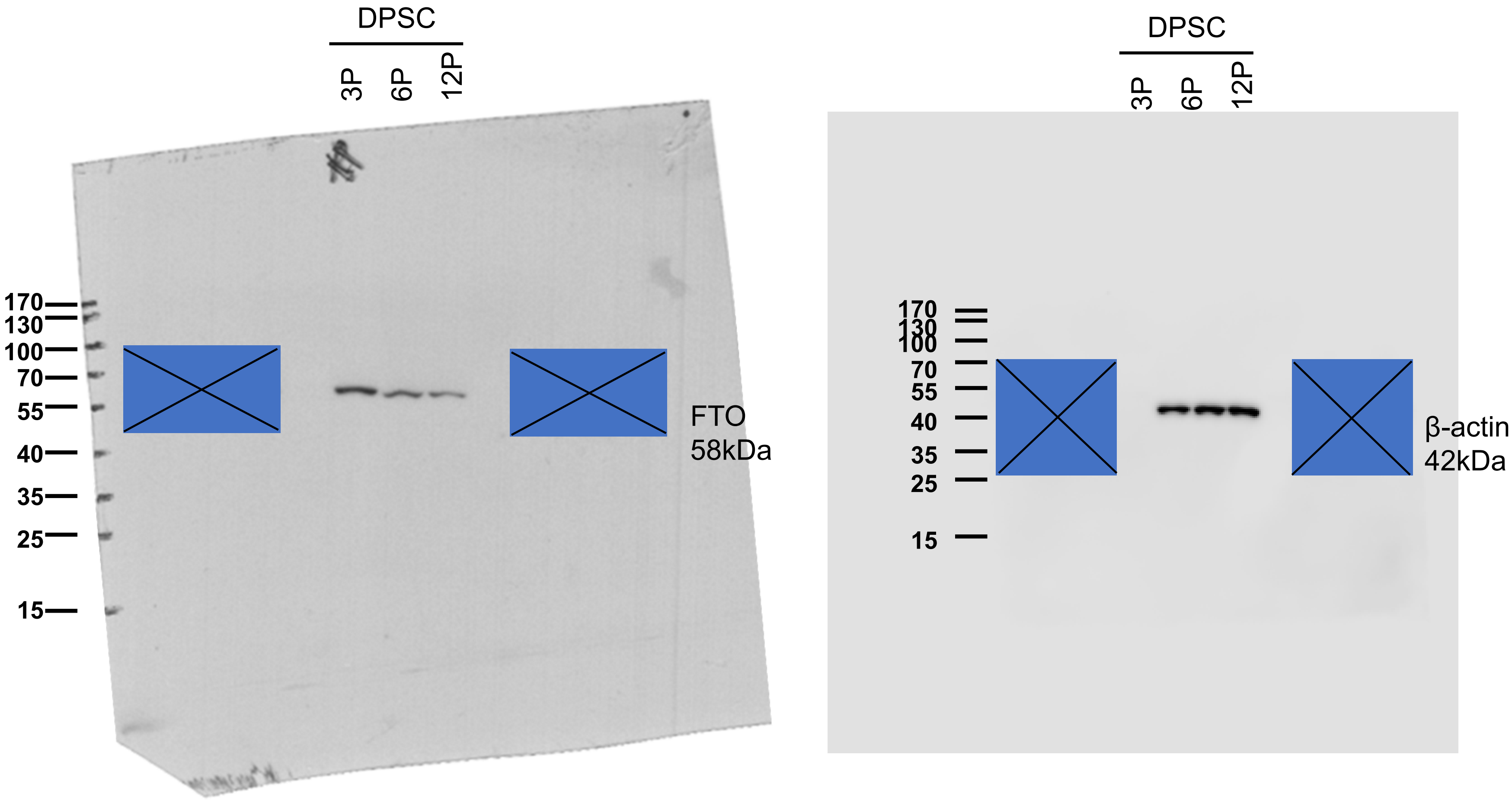

Figure S2.Original Western blot image of Figure1D.

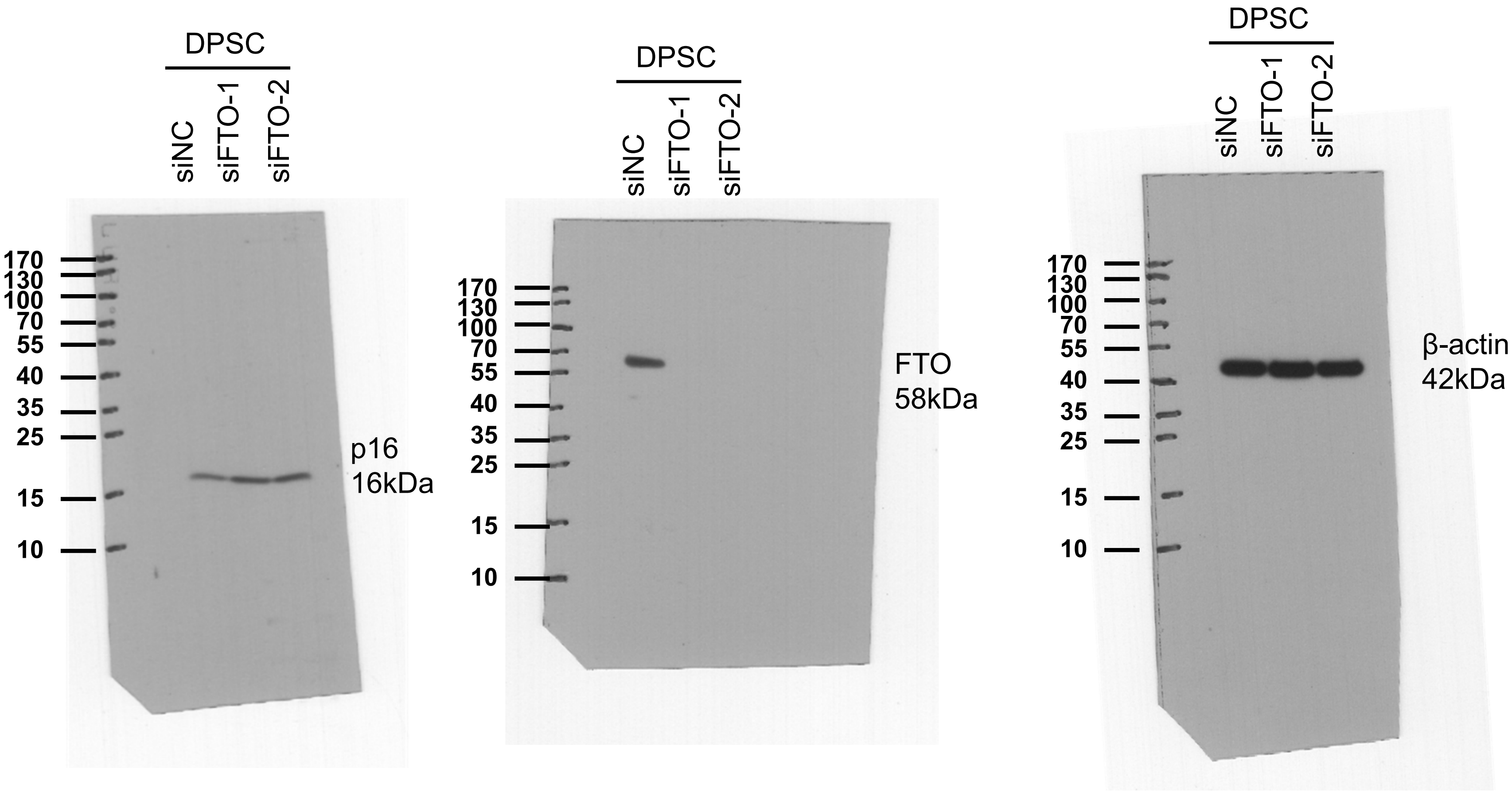

Figure S3.Original Western blot image of Figure 2B.

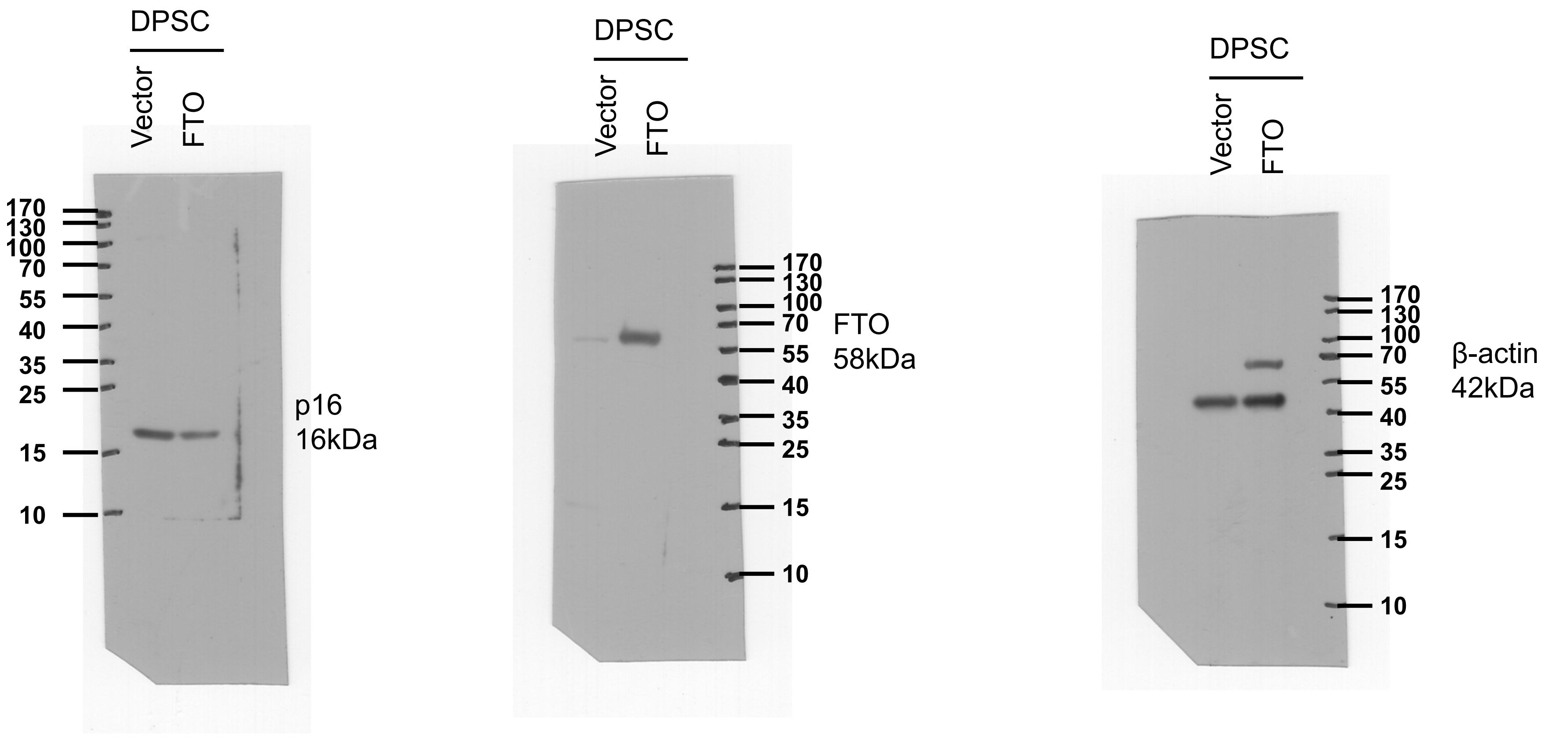

Figure S4.Original Western blot image of Figure 2D.

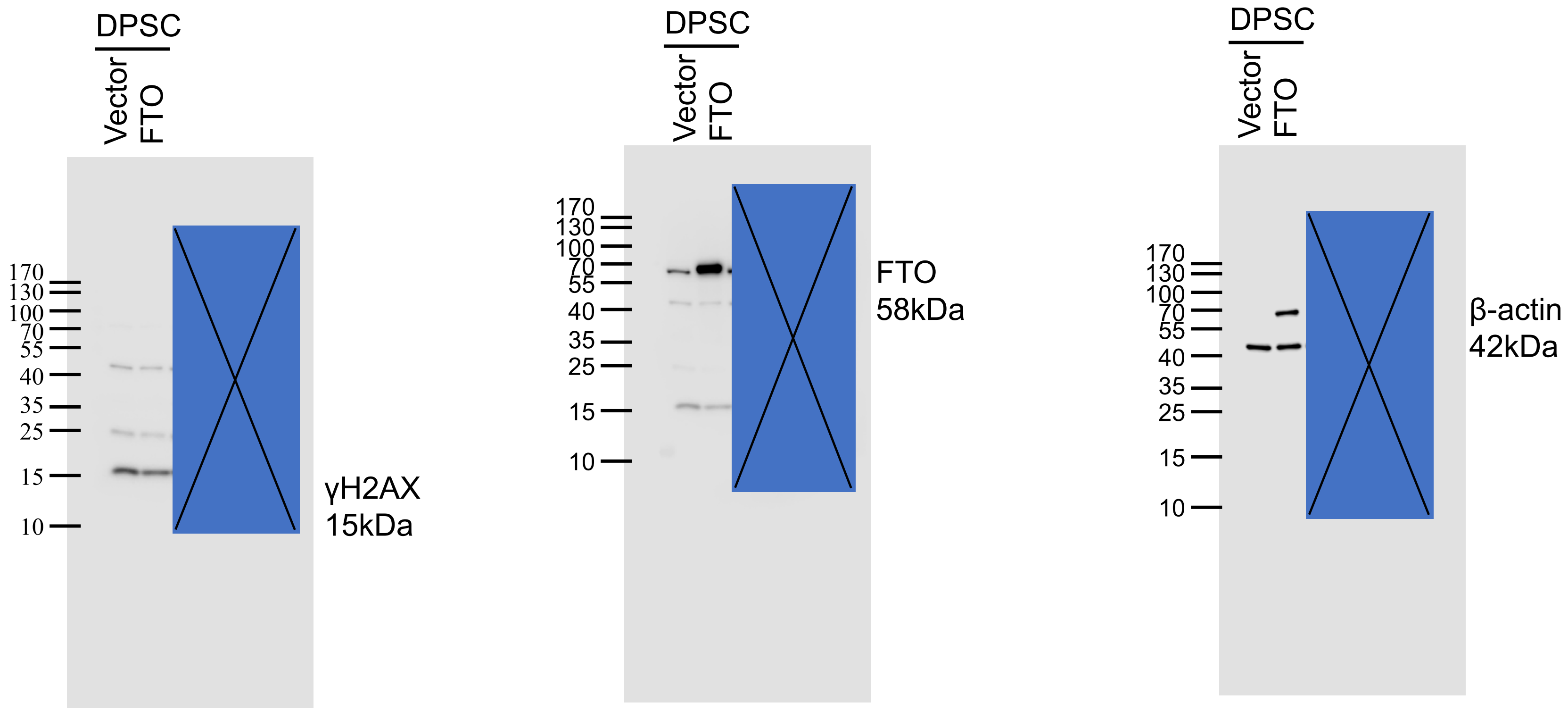

Figure S5.Original Western blot image of Figure 3E.

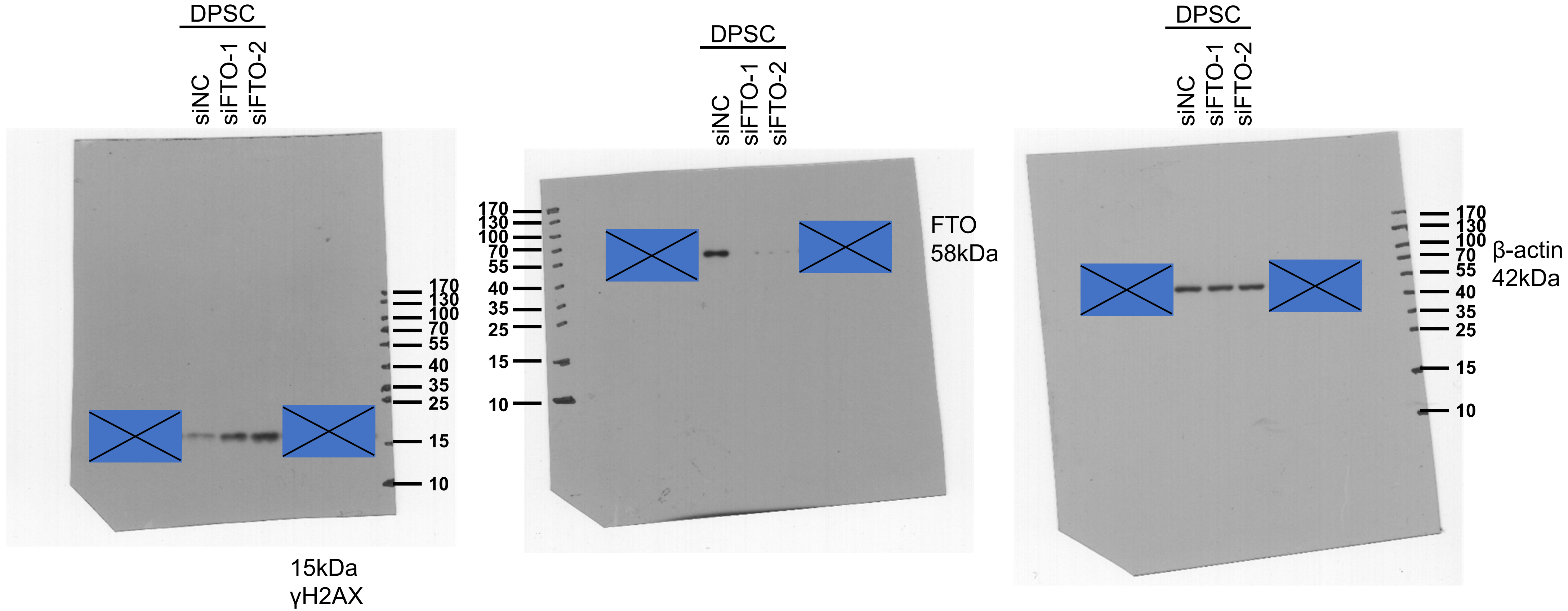

Figure S6.Original Western blot image of Figure 3G.

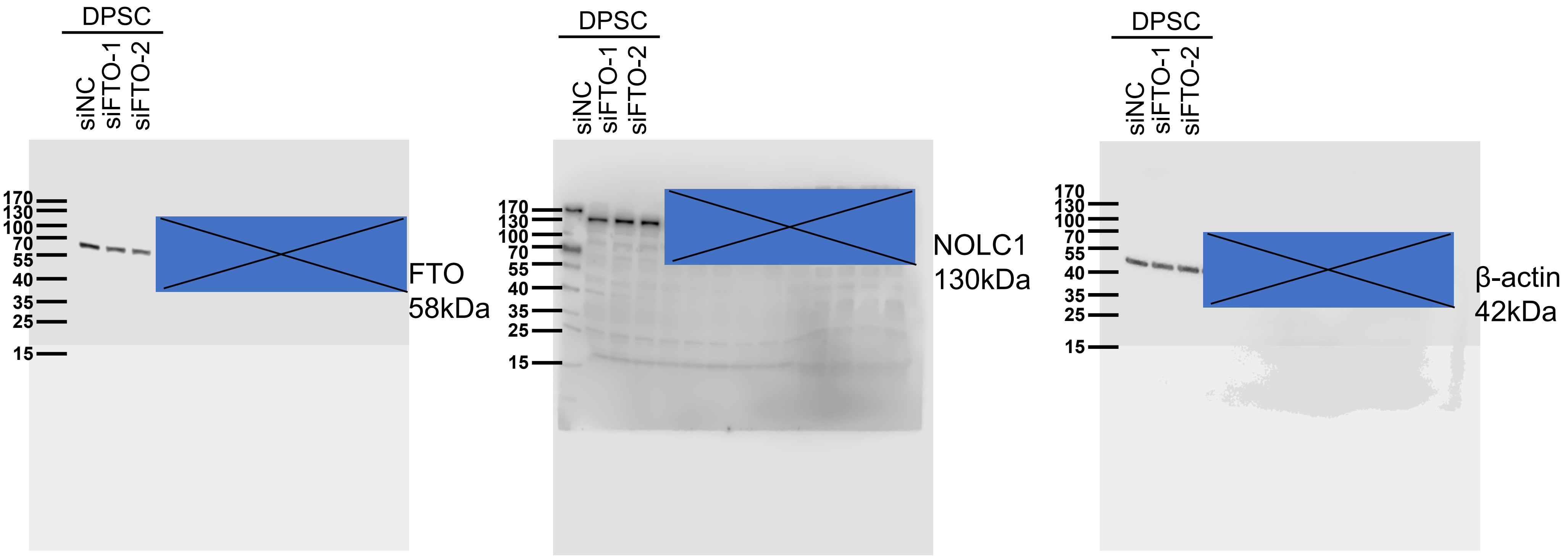

Figure S7.Original Western blot image of Figure 4H.

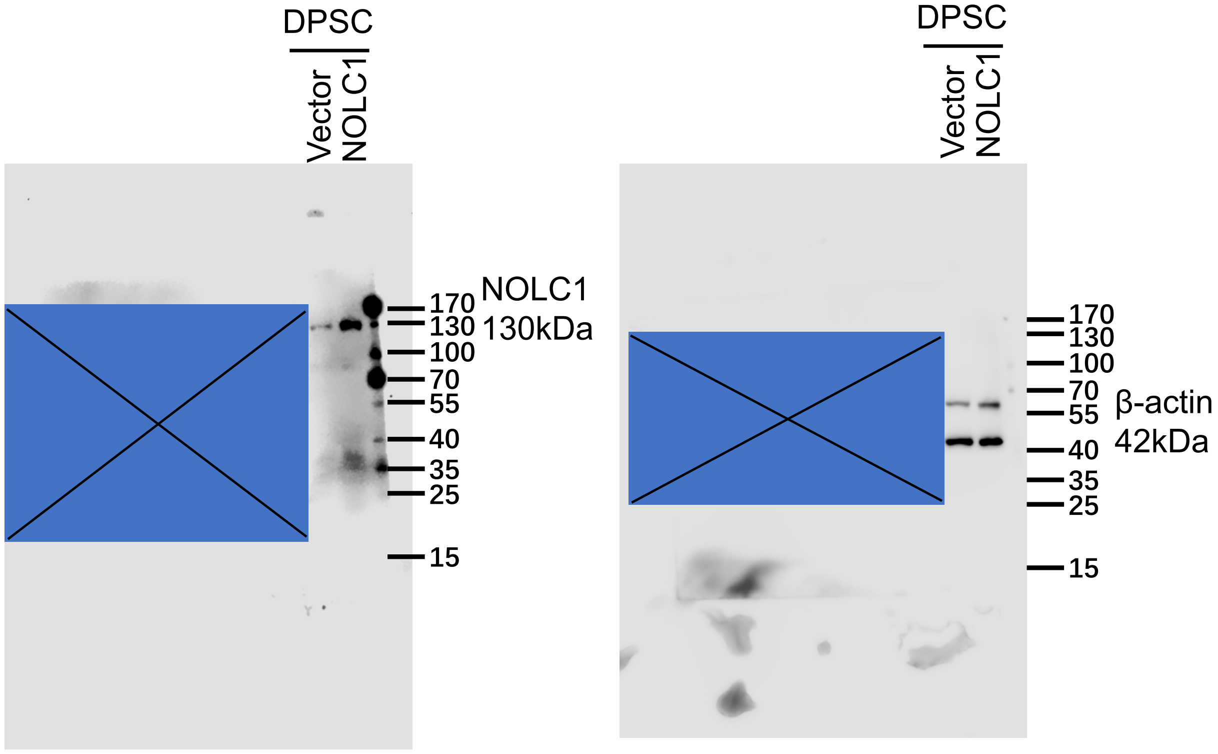

Figure S8.Original Western blot image of Figure 4J.

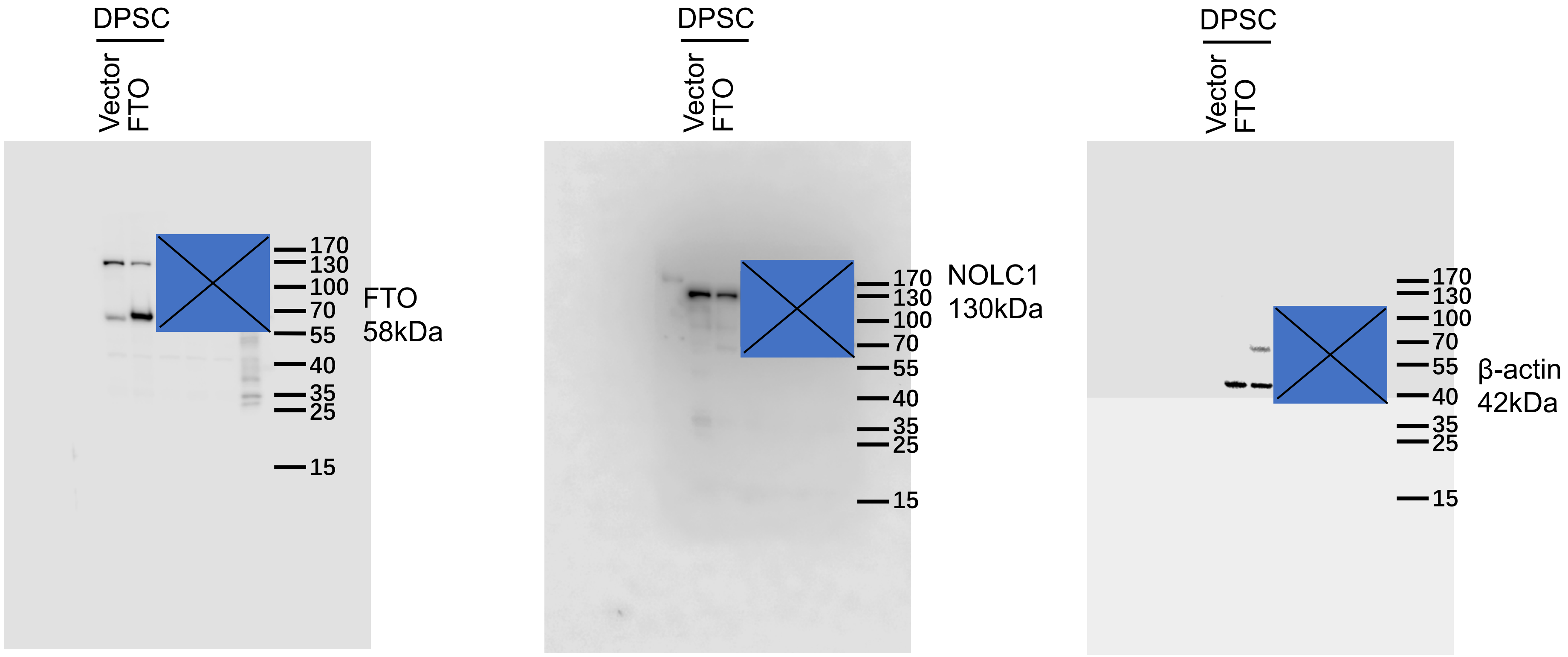

Figure S9.Original Western blot image of Figure 4M.

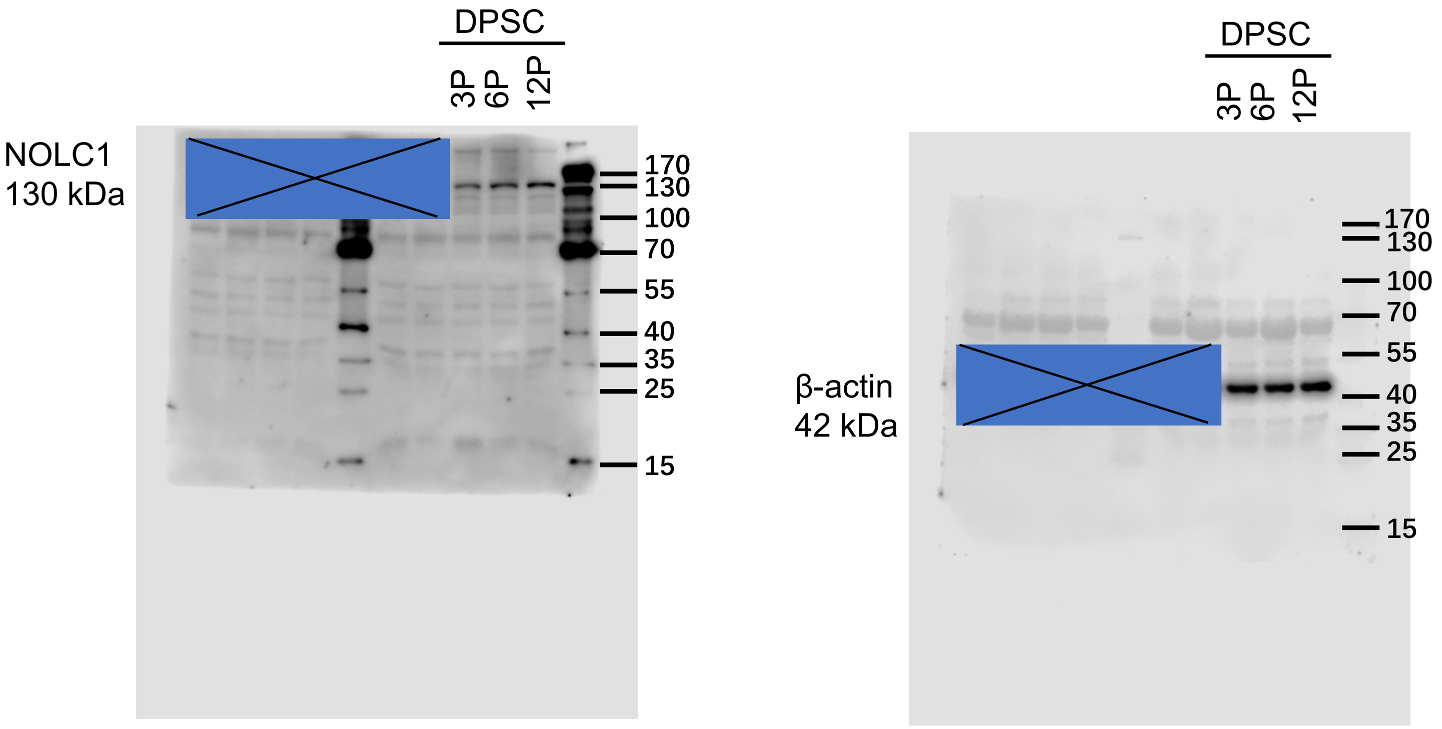

Figure S10.Original Western blot image of Figure 5B.

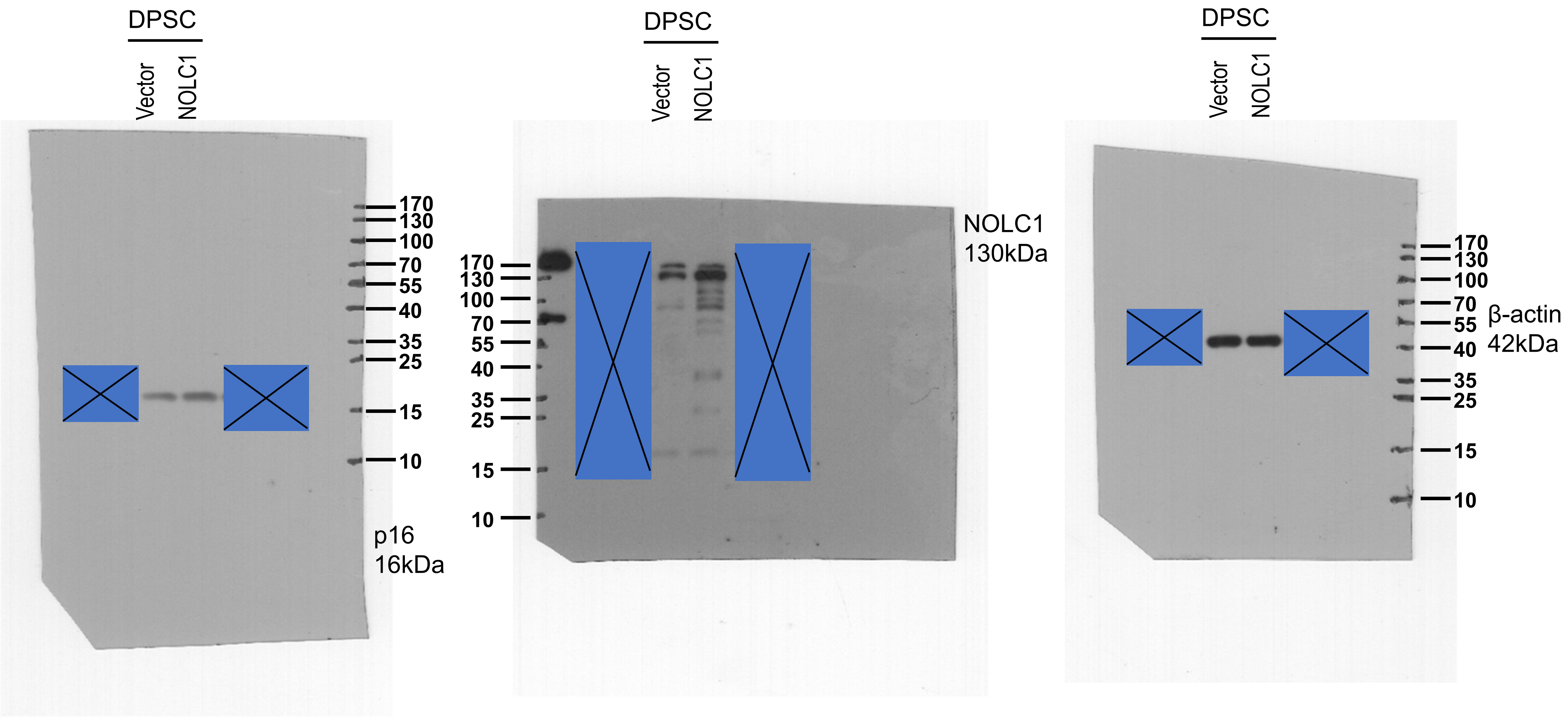

Figure S11.Original Western blot image of Figure 5C.

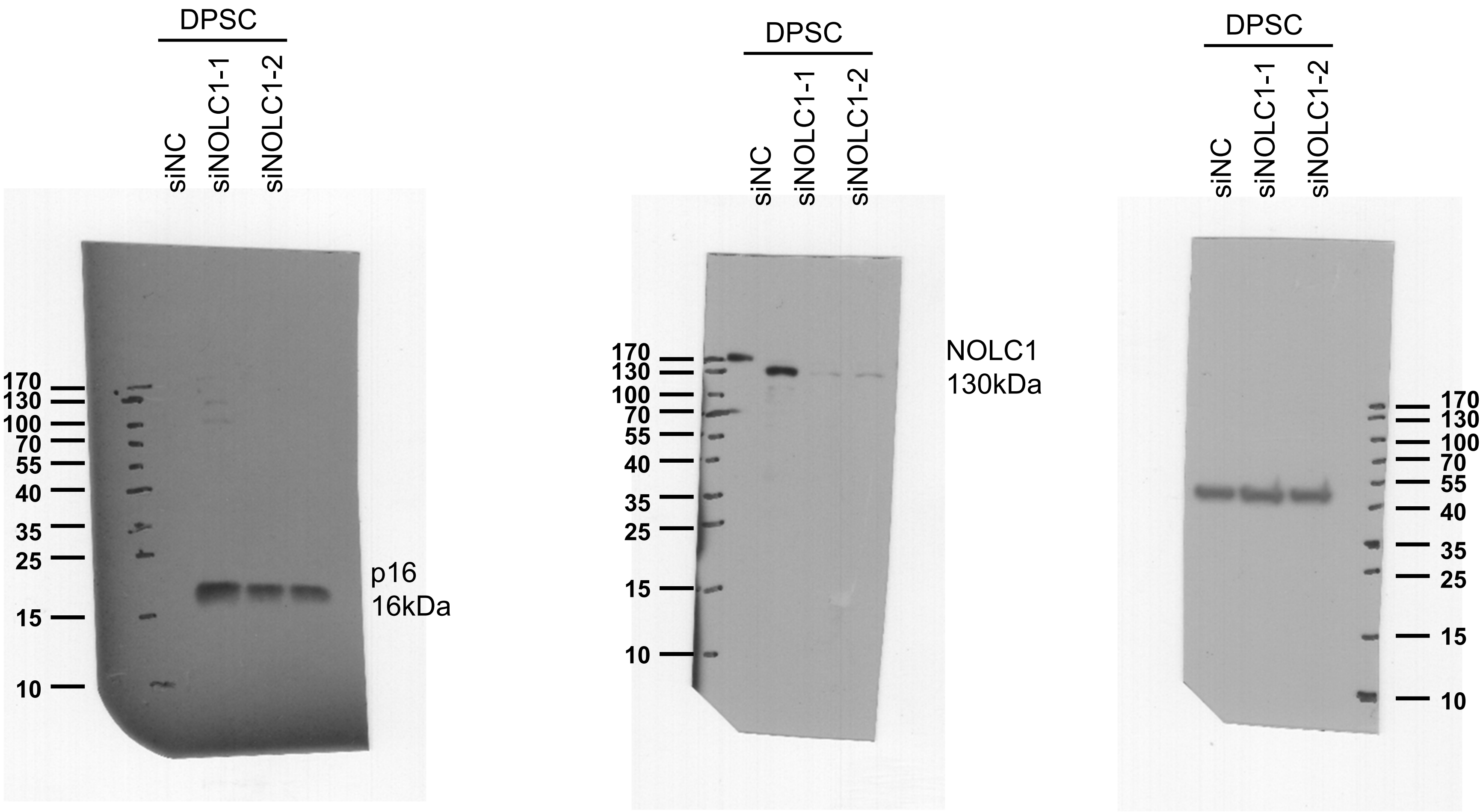

Figure S12.Original Western blot image of Figure 5E.

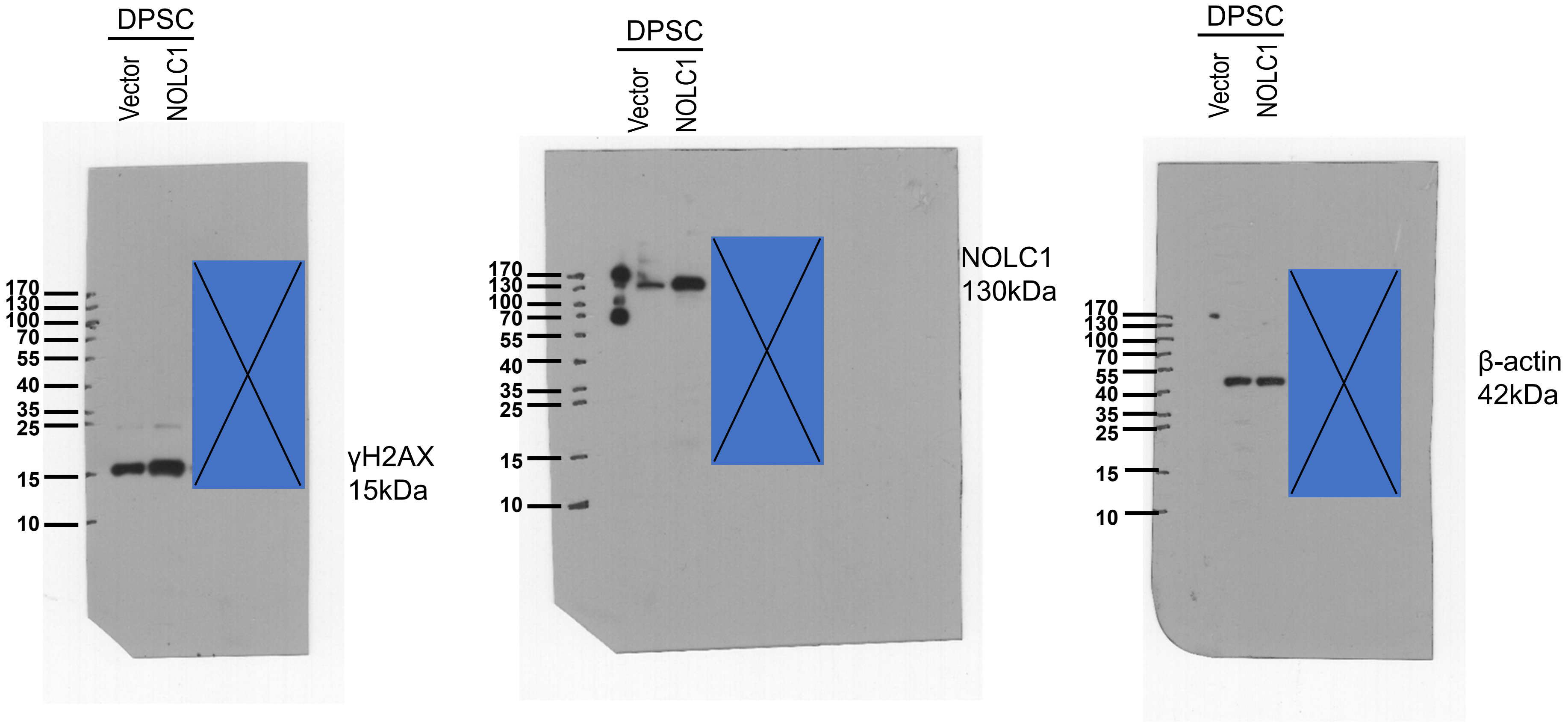

Figure S13.Original Western blot image of Figure 5I.

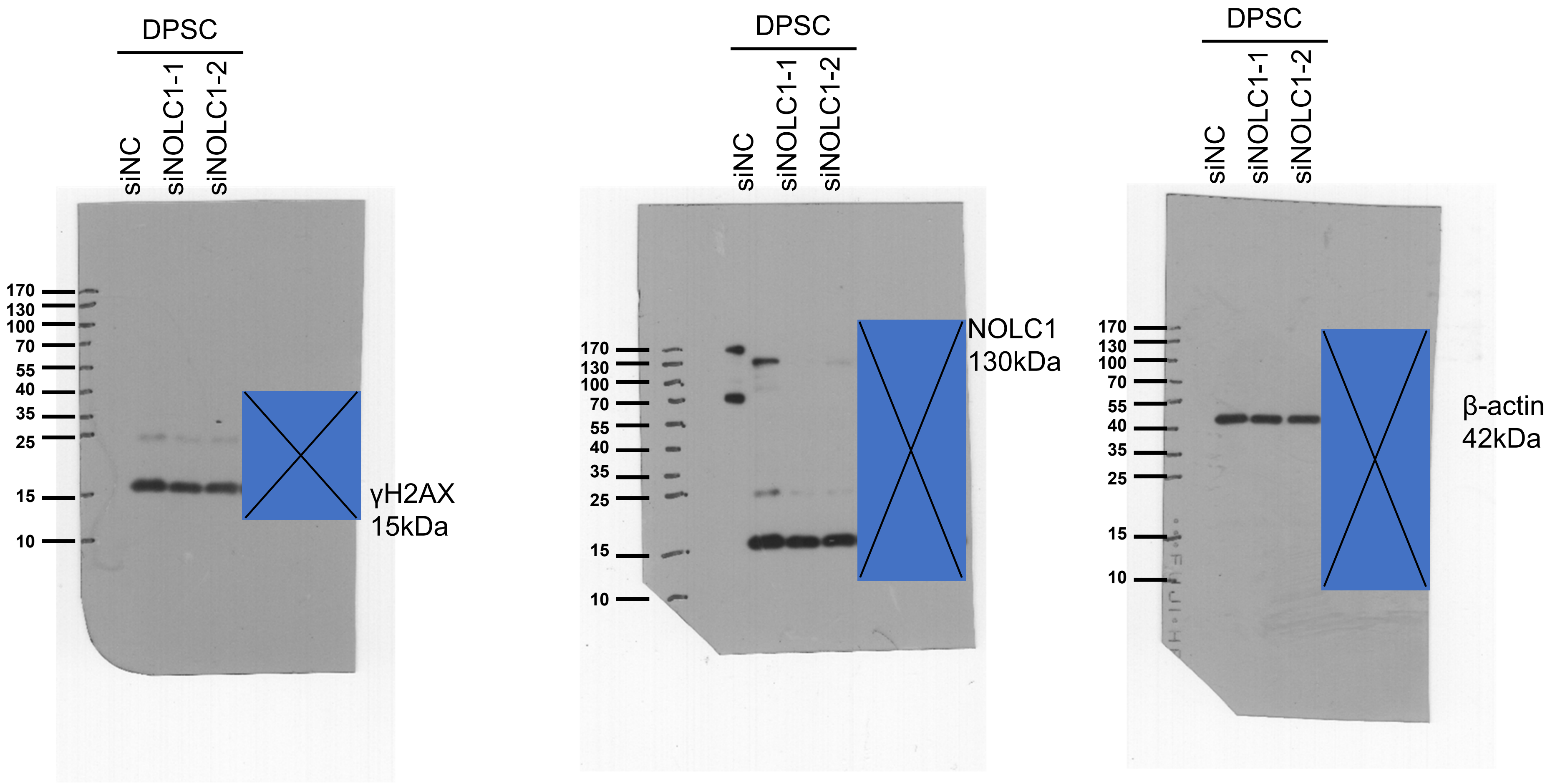

Figure S14.Original Western blot image of Figure 5K.

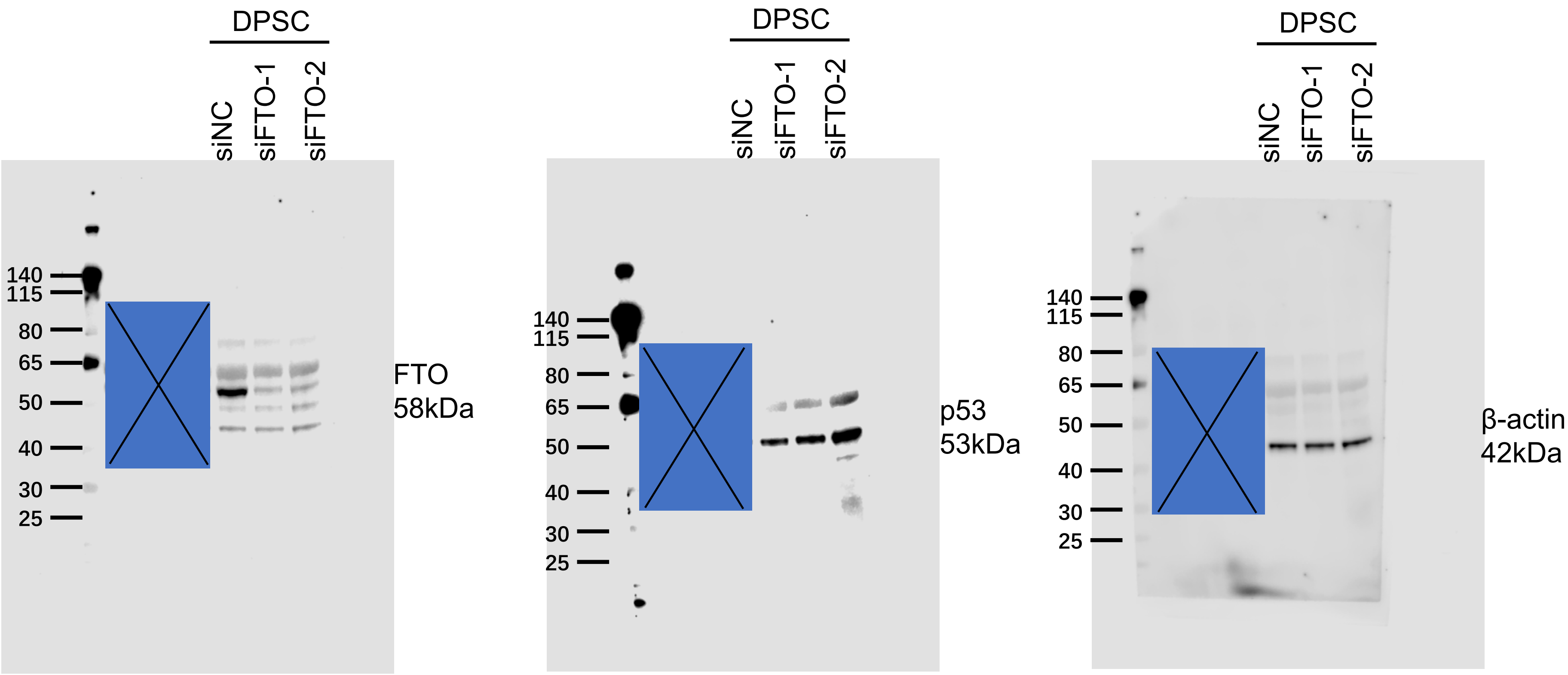

Figure S15.Original Western blot image of Figure 6A.

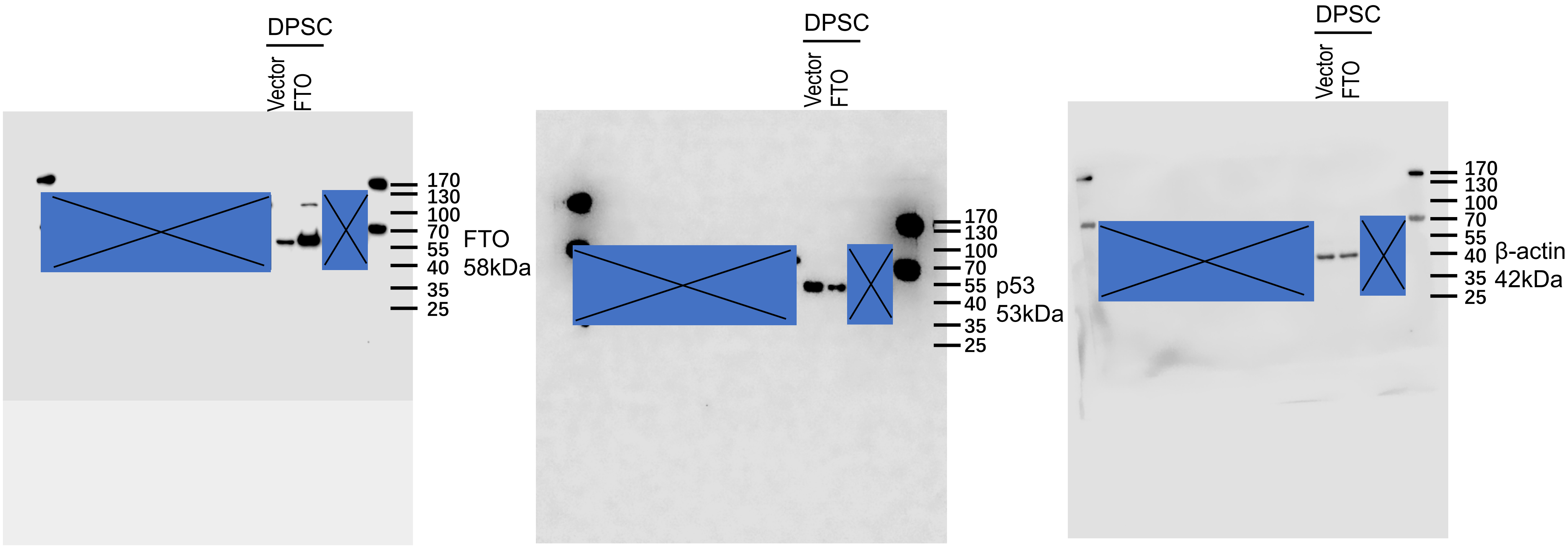

Figure S16.Original Western blot image of Figure 6B.

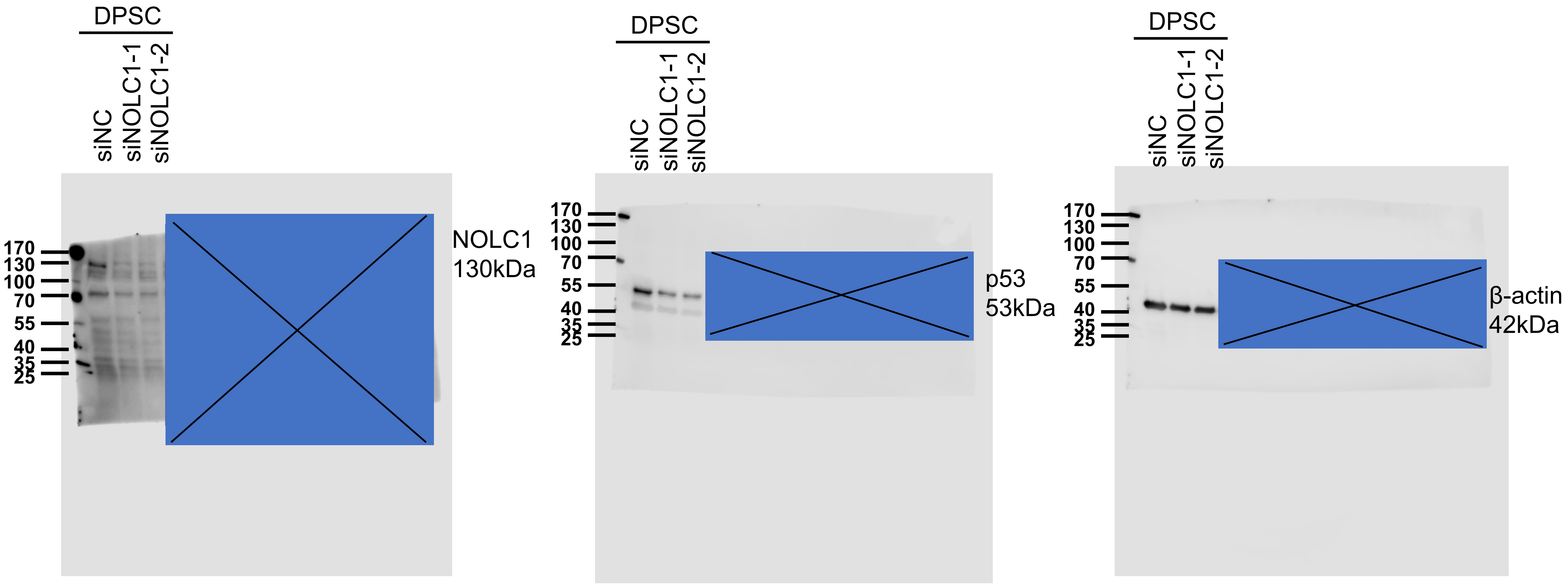

Figure S17.Original Western blot image of Figure 6C.

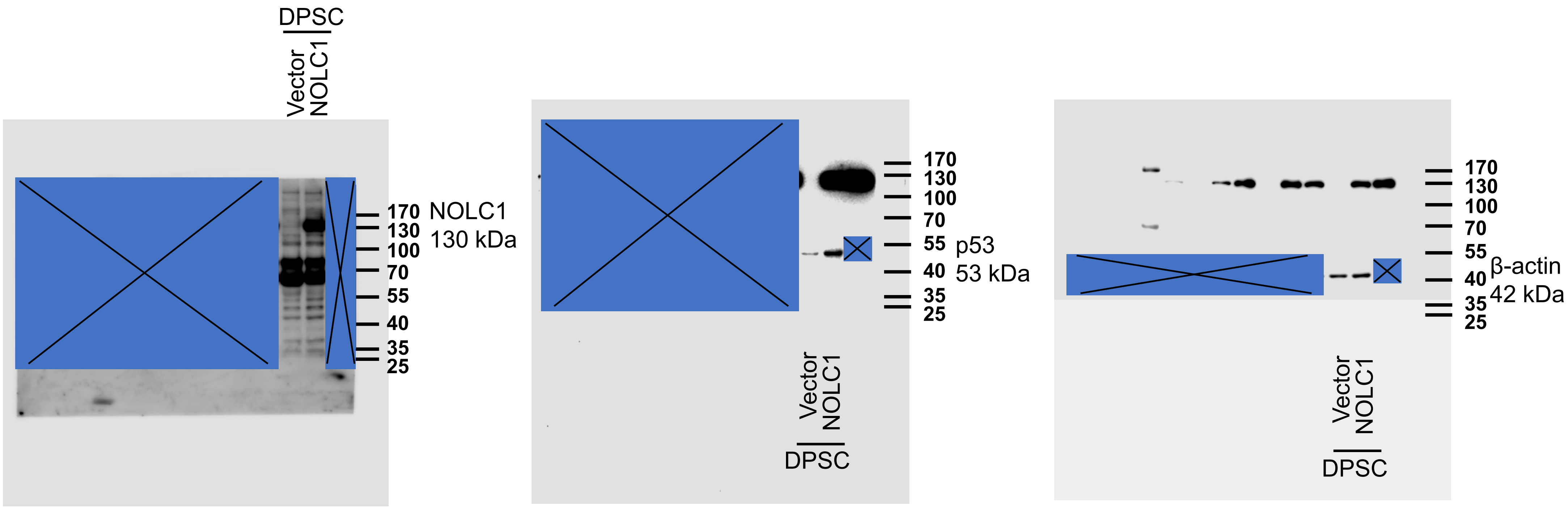

Figure S18.Original Western blot image of Figure 6D.

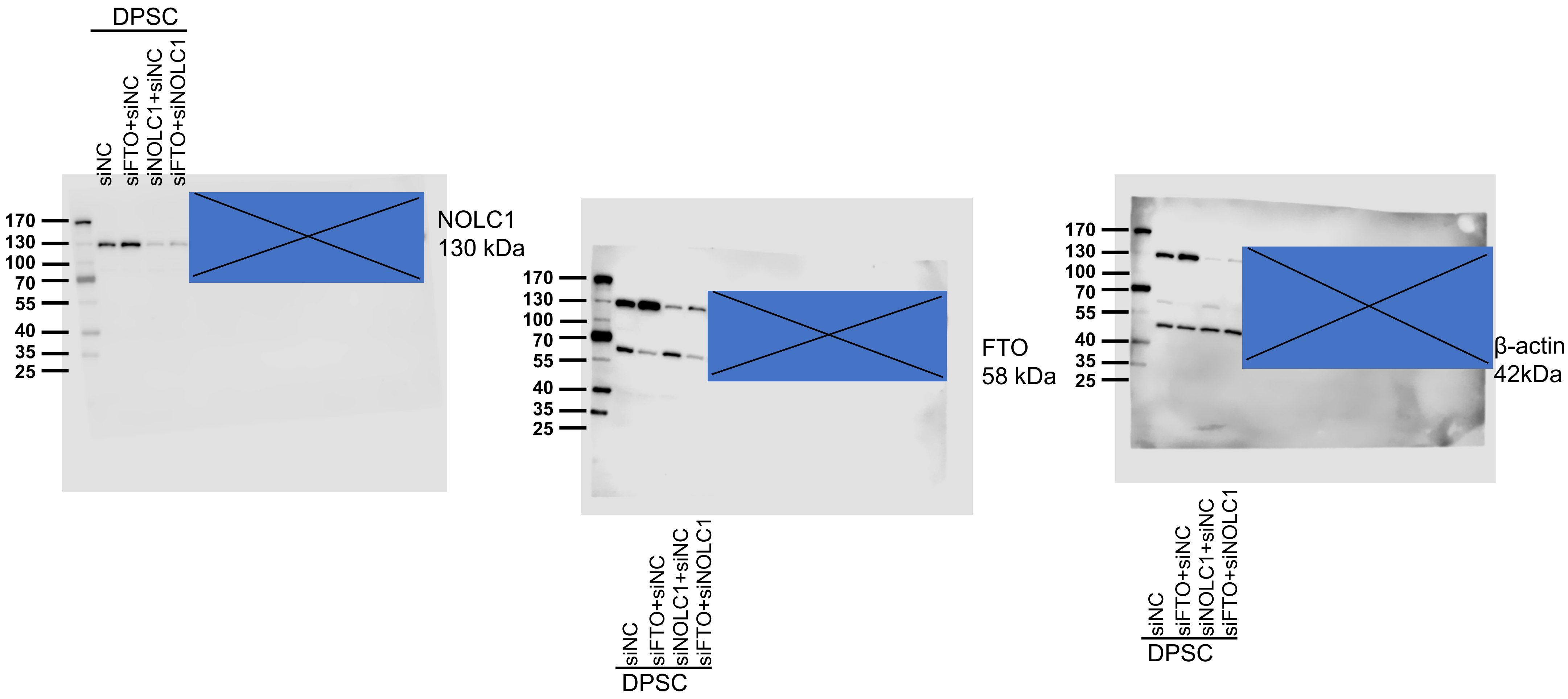

Figure S19.Original Western blot image of Figure 7H.

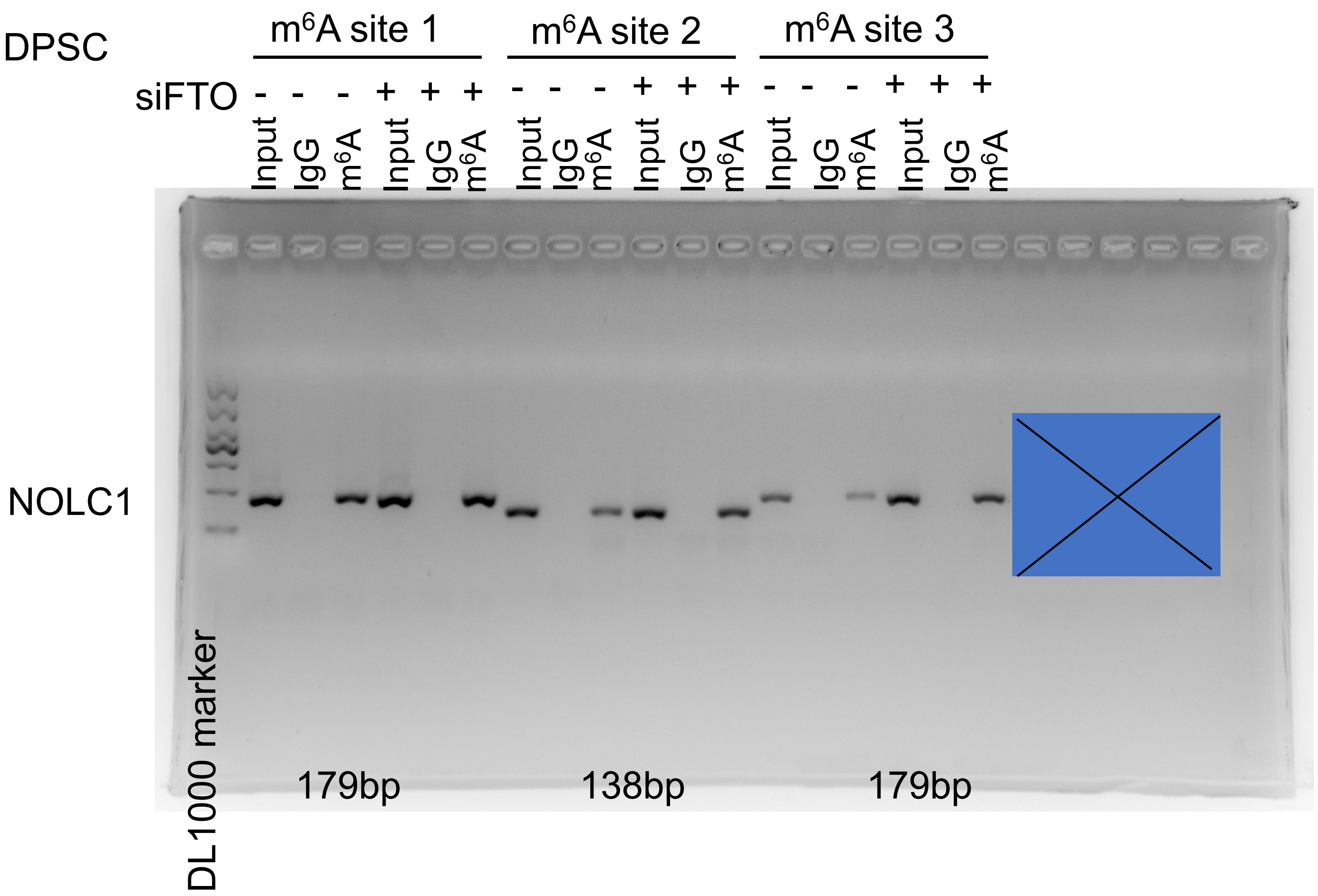

Figure S20. Original RT-PCR image of Figure 7A.

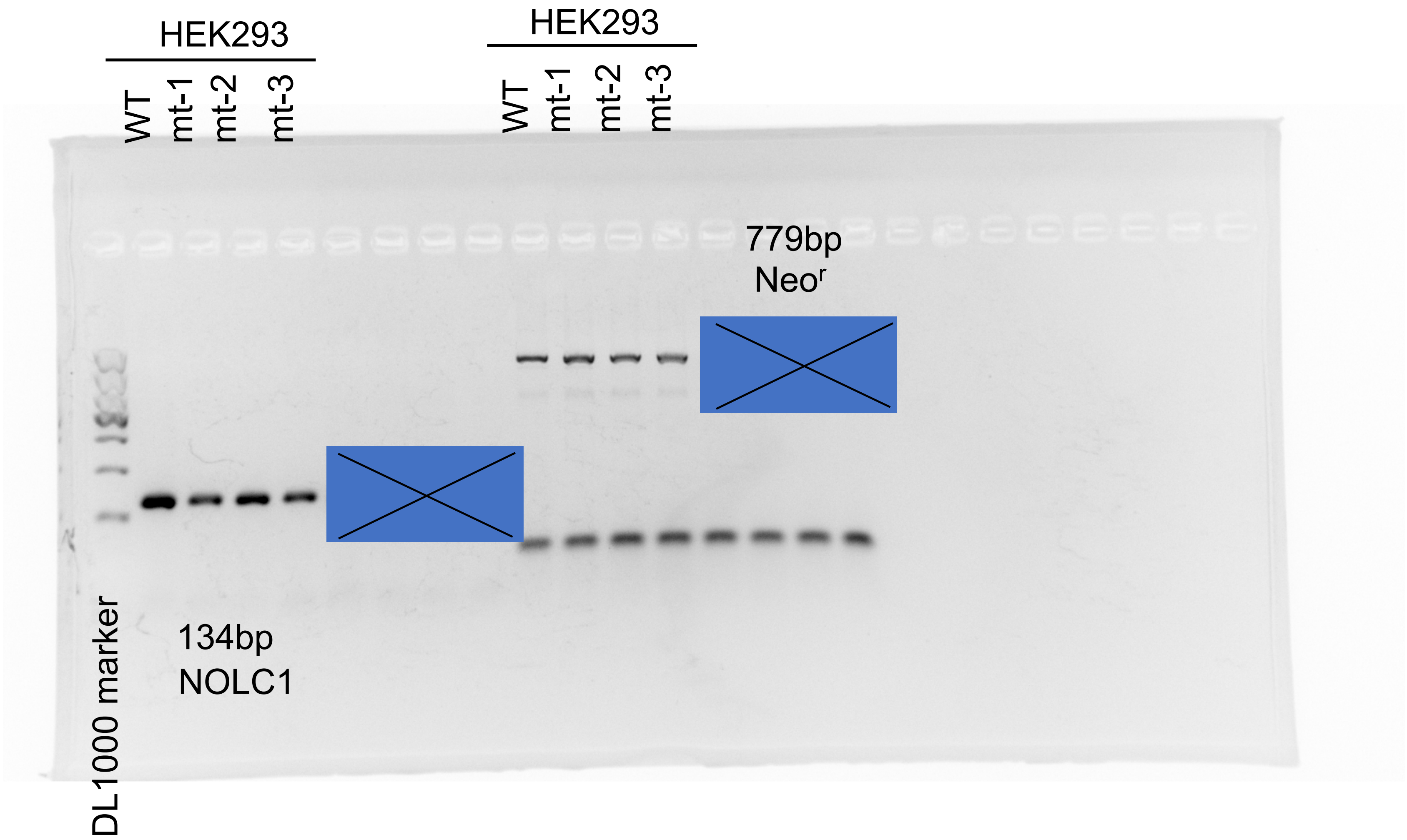

Figure S21. Original RT-PCR image of Figure 7C.
